# Metadata Stewardship in Nanosafety Research: Community-Driven Organisation of Metadata Schemas to Support FAIR Nanoscience Data

**DOI:** 10.3390/nano10102033

**Published:** 2020-10-15

**Authors:** Anastasios G. Papadiamantis, Frederick C. Klaessig, Thomas E. Exner, Sabine Hofer, Norbert Hofstaetter, Martin Himly, Marc A. Williams, Philip Doganis, Mark D. Hoover, Antreas Afantitis, Georgia Melagraki, Tracy S. Nolan, John Rumble, Dieter Maier, Iseult Lynch

**Affiliations:** 1 School of Geography, Earth and Environmental Sciences, University of Birmingham, Birmingham B15 2TT, UK; 2 Novamechanics Ltd., 1065 Nicosia, Cyprus; afantitis@novamechanics.com (A.A.); Melagraki@novamechanics.com (G.M.); 3 Pennsylvania Bio Nano Systems, Doylestown, PA 18901, USA; fred.klaessig@verizon.net; 4 Edelweiss Connect, CH-4057 Basel, Switzerland; Thomas.Exner@edelweissconnect.com; 5 Department of Biosciences, Paris Lodron University of Salzburg, 5020 Salzburg, Austria; sabine.hofer2@stud.sbg.ac.at (S.H.); norbert.hofstaetter@stud.sbg.ac.at (N.H.); Martin.Himly@SBG.AC.AT (M.H.); 6 U.S. Army Public Health Center (APHC), Aberdeen Proving Ground—South, Aberdeen, MD 21010, USA; usarmy.apg.medcom-phc.mbx.tox-info@mail.mil; 7 School of Chemical Engineering, National Technical University of Athens, 157 80 Athens, Greece; fdoganis@chemeng.ntua.gr; 8 Mark D Hoover LLC, Morgantown, WV 26505, USA; markdhoover@comcast.net; 9 Department of Biomedical Informatics, University of Arkansas for Medical Sciences, Little Rock, AR 72205, USA; TNolan@uams.edu; 10 R&R Data Services, Gaithersburg, MD 20877, USA; jumbleusa@earthlink.net; 11 CODATA-VAMAS Working Group on Nanomaterials, 75016 Paris, France; 12 Biomax Informatics AG, 82152 Planegg, Germany; dieter.maier@biomax.com

**Keywords:** nanosafety, (meta)data, data curation, data management roles, data shepherd, FAIR scientific principles

## Abstract

The emergence of nanoinformatics as a key component of nanotechnology and nanosafety assessment for the prediction of engineered nanomaterials (NMs) properties, interactions, and hazards, and for grouping and read-across to reduce reliance on animal testing, has put the spotlight firmly on the need for access to high-quality, curated datasets. To date, the focus has been around what constitutes data quality and completeness, on the development of minimum reporting standards, and on the FAIR (findable, accessible, interoperable, and reusable) data principles. However, moving from the theoretical realm to practical implementation requires human intervention, which will be facilitated by the definition of clear roles and responsibilities across the complete data lifecycle and a deeper appreciation of what metadata is, and how to capture and index it. Here, we demonstrate, using specific worked case studies, how to organise the nano-community efforts to define metadata schemas, by organising the data management cycle as a joint effort of all players (data creators, analysts, curators, managers, and customers) supervised by the newly defined role of data shepherd. We propose that once researchers understand their tasks and responsibilities, they will naturally apply the available tools. Two case studies are presented (modelling of particle agglomeration for dose metrics, and consensus for NM dissolution), along with a survey of the currently implemented metadata schema in existing nanosafety databases. We conclude by offering recommendations on the steps forward and the needed workflows for metadata capture to ensure FAIR nanosafety data.

## 1. Introduction

As long-term historical data on the effects of engineered nanomaterials (NM) are lacking, the extensive use of NM in everyday life raises questions regarding their fate, the environmental and biological exposure routes and the potential risks for the environment, biological organisms, and humans [1,2]. A key challenge for the risk assessment of NM is their dynamic nature, whereby NM may transform during storage [3,4], in product formulations [5], and when released and consequently exposed to media or living organisms [6,7]. Regulatory frameworks, such as the Registration, Evaluation, Authorisation and Restriction of Chemicals (REACH) in Europe and the Toxic Substances Control Act (TSCA) in the United States, were instituted prior to the widespread application of nanotechnologies and therefore did not differentiate between nanomaterials and their larger, bulk forms. Recognising these differences [8,9], the European Chemicals Agency (ECHA) developed and revised several guidance documents outlining nano-specific considerations to be accounted for by companies registering chemicals (registrants) that came into force in January 2020 [10]. Specifically, the new guidance introduces the concept of “nanoforms” and sets of nanoforms, and requires registrants to justify the basis on which NMs are grouped. A similar situation is present in the United States where TSCA underwent some revisions when amended in 2016 [11]. These revisions included rules on the information gathering of existing and the premanufacture notifications for new NMs. For existing NMs, the U.S. Environmental Protection Agency (U.S. EPA) requires information on the chemical identity, the manufacturing methods, and production volumes, processing, use, ecological and human exposure, release information, and available health and safety data.

Besides these changes in regulation, there is ongoing and intensifying public and legislative pressure to move toxicology, risk assessment and most importantly, for this paper, nanosafety research towards the 3Rs principles (replacement, reduction, and refinement) and to reduce over-reliance on in vivo experiments in the absence of sound scientific justification. Taking into account the additional complexity of NMs compared to small molecules, e.g., NMs require more information to be described like core chemistry, coating functionalisation, while small molecules can be described just by their structure, there are many remaining challenges regarding test methods, interpretation, standardisation and validation [12] including provisions for harmonisation and validation of protocols through the Organisation for Economic Co-Operation and Development (OECD) [13,14]. Alternative testing strategies (ATS), also termed new approach methodologies (NAMs), are based on in vitro models and the emergence of nanoinformatics has been suggested as a way to incorporate advancing scientific knowledge into regulation [15,16]. In most cases, NAMs give important insights into the mechanism of NM interactions with a biological environment suspected of, or known to provoke adverse effects and/or how NM and system are transformed by these interactions. However, each NAM by itself is unable to fully characterise an NM’s state and predict directly a toxicological endpoint, as this would require NAMs to be combined into test batteries based on integrated approaches to testing and assessment (IATAs), next generation risk assessment (NGRA), and a phased approach to toxicological testing [17,18]. The design, improvement, and production of ‘safer’ NMs (safer-by-design) will thus ultimately rely on developing a suite of in vitro models [19] and to a progressively lesser extent on in vivo studies [20], supported by validated in silico approaches (computational modelling, quantitative structure-activity relationships (QSARs), and machine learning tools) [21,22,23,24]. These will enable a comprehensive risk assessment that is timely and cost-effective while addressing the significant ethical considerations surrounding animal testing. To support uptake of such models, the OECD has published guidance (ENV/JM/MONO(2007)2) on the steps required to validate a QSAR model so it can be used for regulatory purposes (see Section 3.1.3 for more details) [25]. As a result, nanosafety research is rapidly transforming into a highly competitive and data intensive field. The shift towards translational research, big data, in silico modelling and machine learning has introduced the need for access to large volumes of high-quality and complete data of different types. This includes physicochemical characterisation at different life stages of the NM, as well as the fate, exposure, and environmental health effects. Among the many challenges this need for accessible data raises, probably the most difficult to overcome is the fact that data come from many methods each at different development and standardisation levels, often using test guidelines not yet adapted for NM.

Bringing nanosafety data together in a harmonised and interoperable way, with the possibility of understanding the specifics of an experimental setup and to evaluate the quality of the data, puts high demands on the documentation of data, its curation, and management. Compared to the foci on data management, open data, data standards, and FAIR principles in the earlier publications in this series [26,27,28,29], here, we refer to specific implementations in the form of metadata completeness and reporting along with the respective data. Metadata and data completeness can assist to monitor technology advancements, to understand differences in the used NMs, their state of fate (ageing, modifications by the environment etc.) and potential experimental setup differences causing discrepancies in the outcome. There are attempts to encourage or even enforce reuse of data facilitated through, e.g., the NanoCommons project or the European Union Observatory for Nanomaterials (EUON) and make the available knowledge publicly available to all stakeholders. This is based on the requirements of the EU’s Open Data policy, which can only succeed through the availability of high quality, trustworthy, and complete data and metadata.

**Figure 1:**
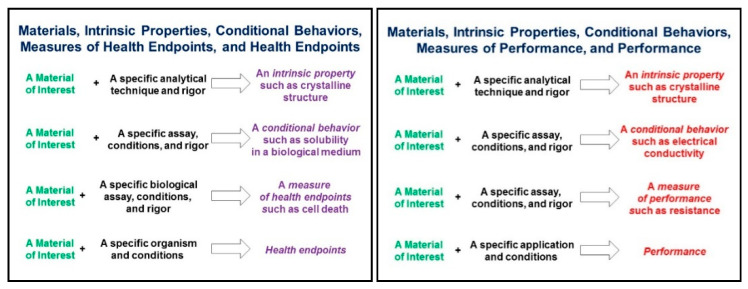
Example study purposes and designs to create data related to health and health endpoints (**left**) and physicochemical or other performance (**right**) for evaluations of the intrinsic properties and conditional behaviours of nanomaterials [30]. Reused with permission from Elsevier, License Number: 4926970512437.

Metadata is defined as data about data, and are necessary to make a dataset meaningful for others. As illustrated in Figure 1, studies to investigate the intrinsic properties and conditional behaviours of nanomaterials can have many purposes and designs, and the purposes and designs of those studies will influence the formulation of appropriate metadata to describe the data that were or will be created [30].

Indexing the metadata makes them findable (much like the table of contents or index in a textbook). Probably, the most well-known metadata capture template is the investigation/study/assay (ISA) tab-delimited (TAB) format, which has been extended to nano also (ISA tab nano) [31,32], although this is not widely implemented by researchers or nanosafety databases in practice. Metadata tools span data warehouses or data upload templates, as well as tools to make data more findable and accessible. Given that tools exist but are not implemented, we will concentrate here on the human factor, either as the contribution of an individual representing a role in the data lifecycle as defined below, or as a community activity or consensus. We hope to highlight that improving the quality of data, handling, and sharing is not the task of just a few individuals developing data warehouses, tools for data handling, and ontologies. Instead, it requires changes in the daily work habits and mentality with respect to the value of data from everyone involved in the generation and analysis of experimental and computational data.

The purpose of this paper is twofold: (1) to structure good data management practices by defining roles along the data lifecycle and their responsibilities, and (2) to provide guidelines and best practices on optimal organisation of data transfer (and metadata) from one role to the next, i.e., from the data producer via the data curators, analysts and data managers to the data user or customers. Another way to frame these questions is how rich metadata, i.e., data describing the data and thus making it understandable to and re-usable by others, should be collected to achieve the goal of providing findable, accessible, interoperable and reusable (FAIR) high-quality data to the community.

A general description of how to improve data quality and FAIRness based on a review of literature and emerging best practices is presented in the first part of the paper. This is complemented by a comprehensive report on the current metadata capturing and reporting practices in nanotechnology and nanosafety research based on responses to a survey distributed amongst stakeholders associated with a range of nanomaterial data resources. The survey and responses can be found in the Supplementary Information focus on how the various data structures address and capture metadata. However, the resources for which respondents agreed to participate in this survey should not be considered exhaustive. Practical illustrations of the importance of metadata are presented as two distinct case studies showcasing how metadata supports data harmonisation, data FAIRness and data extrapolation between fields. The first case study addresses dose metrics and the prediction of NM agglomeration during inhalation in order to determine a delivered dose to correlate with observed toxicity, while the second case study considers the need for agreed terminology as a means to understand the role of NM dissolution as a driver of NM toxicity. Finally, a detailed workflow on the best practice for metadata recording and subsequently FAIRification of nanosafety-aligned data is proposed, and interoperability through detailed metadata reporting is promoted. Adopting this workflow within the nanosafety community will strengthen an ability to combine and create bigger, enriched datasets, link together different existing data sources and provide common search, browsing and accessing functionality, as showcased in the Horizon 2020 NanoCommons project. This will finally facilitate the creation of a scientific digital single market as envisioned, for example, by the European Open Science Cloud (EOSC), by removing scientific and technical barriers hindering data access and re-use, which in turn will maximise the added value of the produced data, reduce the need for new (animal) experiments, promote data-driven innovation, speed up the development of novel safer nano-enabled products, and increase the positive socio-economic impact from scientific research.

### 1.1. FAIRification: Science, Library Science or Both?

This paper is the latest in a series [26,27,28,29] of articles planned by the National Cancer Institute (NCI) Nanotechnology Working Group (Nano WG) Nanomaterial Data Curation Initiative (NDCI) dealing with the emerging field of nanoinformatics and the need for high quality data. The overall objectives of the NDCI include:

- capturing a snapshot of current NM data and metadata curation practices and issues,
- the development of recommendations for moving the nanoinformatics community toward increasingly standardised curation practices, and
- the facilitation of collaborations between researchers, product developers, and others working with NMs that establish and utilise common datasets for cross-boundary work (e.g., application of data from academic institutions to NM product development in industry).

The previous papers addressed the various aspects leading to high quality datasets as described in detail by Hendren et al. [27] and focussed on nanoscience curation workflows [26] and evaluation of the quality and completeness of curated data [28]. In fact, survey results in Karcher et al. (2018) around database integration further outlined the importance of metadata in that integration [29]. In all cases, the need for (meta)data completeness was stressed as a necessity in order to be able to reconstruct the exact experimental setup and methods for improved reproducibility, for evaluating data quality and its combinatorial and interoperable alignment and relevance with data from different sources.

Shortly after the paper by Marchese et al. [28] was published, the FAIR principles [33] were introduced including a set of guidelines for improving the existing infrastructure that supports the reusability of scholarly data. There are two facets to the FAIR principles, one being those issues associated with the technical requirements that data(base) managers must attend to if users are to be able to recall the datasets, and the other being actions that the scientists, and the subject matter experts, must address if their work is to be considered credible by other users. The first facet overlaps with library science, or more specifically, library and information science, and similar issues are encountered when finding a book in a library, i.e., call numbers, subject accession numbers, and subject headings. In library science, the curator’s role includes acquisition, curation (selection of books and deciding when to discard them), archiving, retrieval and circulation for the purpose of dispersing knowledge. Libraries are experienced in annotating (e.g., PubMed’s searchable Medical Subject Headings or MeSH) and there is even the concept of precision and recall as a metric for library effectiveness. Precision and recall measure the retrieval of information in terms of recovering all of the books on a subject without returning too many that are not of interest (see Arora et al. [34] for a nanotechnology database example). These elements certainly contribute to FAIR, but they are more likely to be considered procedural, administrative, and technical relative to the substantive, subject-matter and scientific considerations found in a dataset. We have elected to use the term ‘technical’ for these purposes, but refer the reader to a series of articles by Dr. Birger Hjørland [35,36,37] for a fuller overview of Library and Information Sciences as it moves from ‘collections to connections.’

The technical nature of the FAIR principles is meant to guide data(base) managers, in data digitisation and storage processes, down a path that would be resolvable for machines and, through them to database users. Topics like persistent identifiers, indexing in searchable resources, defined vocabulary, communications protocol and licenses are clearly covered in the FAIR principles, while metadata are more loosely defined as they must meet domain-relevant community standards. In the following sections, we will refer to principles describing features, which need to be implemented by the database or search/browsing services, and those associated with library science and which are subject domain-independent, as technical, while features such as subject domain concepts and standards, especially metadata completeness, that need to be defined by the specific community, as scientific. The only non-technical guidance on the (meta)data completeness and (re)usability is that the metadata need to be sufficiently well-described and rich that they can be automatically (or with minimal human effort) linked or integrated, like-with-like, with other data sources [33]. Defining what completeness means in a specific community is only the first step since, science is continuously progressing and data becomes more interlinked over the different disciplines and communities, but does not provide guidance on how scientific information should be translated into metadata. Such guidance needs to originate from people with sufficient knowledge of the experimental practices but also with an understanding of data or library science, who essentially liaise between the two disciplines, and shepherd the data creators (experimentalists, computational scientists) through the processes, by raising awareness of their responsibilities as data creators and the need for active participation in the definition and validation of metadata coverage, as discussed later in this paper.

Although the original “technical” FAIR principles are mainly focussed on how data should be shared [33], and not on what needs to be reported to make the data reusable with high confidence, the authors were very successful in raising concerns about the amount and variability of shared data. As a result, FAIRness, FAIRification (the process of making data FAIR), and FAIR measures have been introduced into requirements for data management plans of all major funding agencies. On the other hand, this highly technical focus could be used as an argument to push the responsibility to achieve data FAIRness to data curators and database managers, which have to develop the access protocols and search engines, and may been seen to absolve data producers of responsibility. Thus, a gap still exists when it comes to the scientific FAIR principles needed to ensure that high-quality data generation and collection, and metadata processing have sufficient “completeness” to facilitate the FAIRification process. Completeness here means the relevant scientific information to ensure that the data are findable in a relevant query and can be understood without needing to ask the scientist involved in its generation for information about the purpose it was generated for, or how it was generated. Therefore, we decided to follow the same structure when drafting the following scientific principles enabling a combined list of principles for both technical and scientific FAIRness.

- To be findable in a scientific context:
  ○ SF1: Use standard, unambiguous identifiers for characterising your samples, test systems and experimental details, presenting as much information as possible. As per the proposals of the GO FAIR Chemistry Implementation Network [38], coordination and some formalisation is needed to promote interoperability between different types. For example, chemistry-aware identifiers like IUPAC names, PubChem compound identifiers, InChIs and the recently proposed NInChIs (nano-InChIs, see Lynch et al. in this special issue) provide orthogonal information to compound/substance names and CAS RN, and the use of both is preferred.
- To be accessible:
  ○ SA1: Annotate metadata and data and especially metadata/data schemas with standardised ontologies to make them computer accessible. It would be desirable to create a “dictionary” of terms regularly used and thus to use persistent ontological IDs for the metadata and data produced.
  ○ SA2: Make sure that the metadata can be accessed from the same resource as the data. If the data warehouse is not flexible enough to provide the scientific metadata through its metadata access functionalities, provide it in a standardised way (either standard file formats like ISA or supplemented by a clear access protocol—see recommendations for further details).
  ○ SA3: Provide your protocols in FAIR resources, in addition to the materials and methods section of the corresponding paper. Remember that your data might be used in another context and exact descriptions are needed. Linking protocols to data via electronic laboratory notebooks is one approach to achieve this.
  ○ SA4: Document small deviations from the original/standardised protocol with your metadata/data. For example, if different samples are using different DMSO concentrations for controlling the production and properties of nanomaterials, this should be reported per sample in contrast to the protocol where only the DMSO range will be given.
- To be interoperable:
  ○ SI1 links to descriptions of the test methods, protocols and quality control measures: Provide direct res to give the user the chance to evaluate data interoperability. In this way, additional information, which cannot all be covered by the metadata can be easily accessed.
  ○ SI2: Report protocol metadata in a structured and annotated way to allow harmonisation and interlinking of data. Even if duplication of information in the protocol and the metadata is sometimes needed or even preferred, guarantee consistency between both.
- To be reusable:
  ○ SR1: Do not limit the reported metadata to fulfil only the requirements of the study for which the data was produced. Sections 6 and 7 provide examples on the usage of data in a different computational context than the experimental initially intended.
  ○ SR2: Establish a feedback loop between data creators, analysts and customers to continuously improve the metadata completeness and quality. Keep in mind that scientific progress can lead to new use cases and go beyond “standards” defined at a specific point of time.

After defining the necessary scientific FAIR principles, the next step is to define and implement the process of how to undertake the (meta)data collection, curation and transferability between stakeholders, and the storage and sharing of data and metadata to maximise quality and FAIRness. To achieve this, we firstly need to identify the mosaic that constitutes the data ecosystem, establish the different roles comprising it and optimise the potential interoperabilities.

### 1.2. Roles and Responsibilities in (Meta) Data Collection, Curation and Accessibility, Their Dependencies and the Need for Data Shepherds

The concept of data completeness as outlined by Marchese et al. [28] is quite abstract and is strongly linked with specific data usage, i.e., use of data for regulatory decision making would have much stricter requirements for data completeness than, for example, utilisation of data for modelling where gaps can be input, or end-points with missing data removed from the input dataset. To progress the FAIRification of nanosafety data, there is a need to define metadata reporting standards (in the sense of guidelines for what information needs be reported rather than as standard templates with defined, fixed metadata fields such as the aforementioned ISA-TAB-Nano templates), and to assign clear roles and responsibilities for metadata creation and reporting in order to transform this concept into a practical action plan. These standards are not meant to be set in stone, and indeed activities to do so failed outside the very restrictive and standardised regulatory field. Instead, they need to be adopted, extended and improved to follow scientific evolution and their implementation requires both mental and behavioural change and the expertise of all involved players.

**Figure 2:**
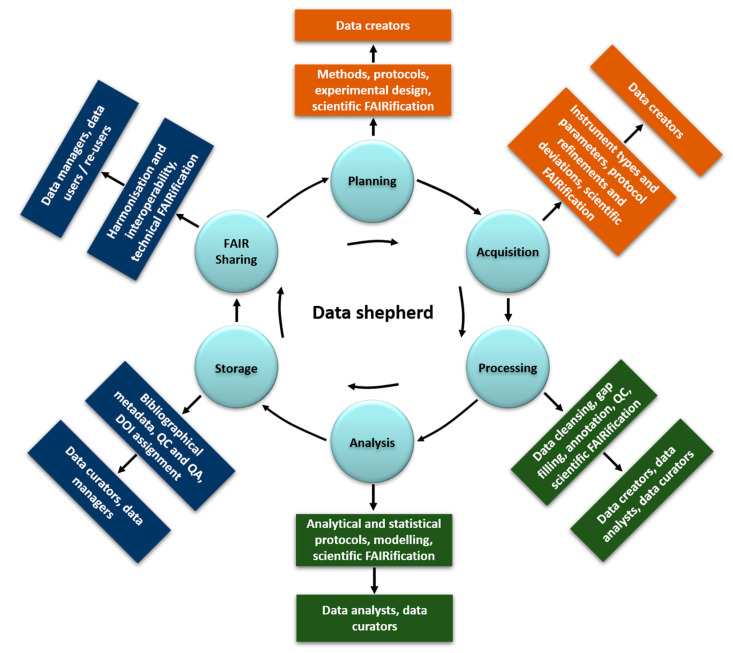
The data lifecycle, the metadata requirements at each stage, and the roles responsible for collecting and reporting the metadata at each step. The data shepherd exists to facilitate communication and streamline the entire workflow. (technical metadata = green, descriptive metadata = orange, bibliographical metadata = navy blue).

Despite its significance, metadata capturing is not widely implemented in everyday academic scientific practice. This is due to lack of training in data management, and the general perception of data management as being something that is done at the end of a project and after the data are fully analysed and published. As a result, data curators accessing data files from others or extracting data from a publication are often unable to locate the information needed to fully implement the necessary metadata. Therefore, the first critical change needed is to shift the design and implementation of data management workflows from project end to project outset. It is imperative to encourage (meta)data upload to data repositories after its creation as depicted in Figure 2. This can be implemented with appropriate access restrictions in place to protect intellectual properties where necessary, and with automatic release data following publication. As a result of the cyclical nature of the data lifecycle, it is always possible to optimise the (meta)data coverage, taking into account the needs of different users and re-users.

Based on the data lifecycle, shown in Figure 2, which starts from experimental design through long term storage and sharing including all the necessary FAIRification steps, six general roles can be identified with respect to metadata capturing and usage:

1. Data customers: requestors, accessors, users, and re-users of the needed or produced data (evaluation of the scientific and technical FAIRification step by testing for the final goal of usability and reusability in real applications)
2. Data creator: the experimentalists planning and generating the data (planning, acquisition, and manipulation in the data lifecycle, scientific FAIRification steps in Figure 2)
3. Data analyst: data handling, manipulation, analysis including modelling (processing and analysis, scientific FAIRification steps in Figure 2)
4. Data curator: data and metadata capturing and quality and completeness control (data manipulation and storage in Figure 2)
5. Data manager: data digitisation in a structured and harmonised format. Metadata implementation and link to data (storage and technical FAIRification steps)
6. Data shepherd: a new role strongly encouraged here, which is defined in detail below, who operates throughout the data lifecycle.

Depending on the practices adopted by a group, unit or larger project, these roles can be quite distinct and geographically dispersed, raising questions of coordination among players. In other cases, they may be collapsed into a small circle of closely situated actors, limiting the possibility for cross-field communication. Nanosafety research ranges from NM synthesis and characterisation, to exposure, fate, hazard, systems biology etc., and data sharing and metadata coverage has to work effectively in such a multidisciplinary environment. The lack of clear processes, uncertainties in responsibilities, and missing coordination often leads to a negligence mentality. In such cases, data collection is postponed until the last moment when some of the data producers have already moved on to other positions and a clear succession plan does not exist to assist with continuous and uninterrupted processes and workflow. Additionally, there is often the expectation that database developers and managers need to take care of data storage and sharing in a form directly and easily usable by the researchers, even if it is not clear what this exactly means. Often, this translates to internal spreadsheet templates that only people within the lab can make sense of, making it really hard to sustain a proper database. Similarly, database managers are expected to provide standard data and metadata templates to guarantee the quality of the data even without the necessary domain-specific scientific knowledge. To overcome this, the establishment of specific and detailed workflows, which include complete data and metadata capture, storage, handling, and analysis linked to the data-related roles 1–5 are necessary. Furthermore, implementation should begin at the experimental design stage, be aligned to existing ontologies and nanosafety schema and linked to distinct tasks and timelines for all of these five data roles (see Figure 2).

In practice, many co-dependencies between these data roles and responsibilities can be identified (Table 1). As conveyed in Table 1, the elements in the data lifecycle are consistent with the definition of nanoinformatics as: (1) the science and practice of setting mission objectives and determining which information is relevant to meeting the safety, health, wellbeing, and productivity objectives of the nanotechnology community; (2) developing and implementing effective mechanisms for collecting, validating, storing, sharing, analysing, modelling, and applying the information; (3) confirming that appropriate decisions were made and that desired mission outcomes were achieved as a result of that information; and, finally, (4) conveying experience to the broader community, contributing to generalised knowledge, and updating standards and training [27,39,40]. In contrast to the scenarios described above, where the database system is responsible for defining the metadata and standard templates for reporting them, the heaviest workload and thus the main responsibilities in the data lifecycle pictured here are divided between data creators and analysts. Data curators and managers subsequently act as the link between them and the data customers. Data curators ensure that the data produced is of sufficient quality and discoverable (annotation) and can effectively answer the objectives set as per the requirements of the data customers. Data managers, on the other hand, ensure data discoverability, accessibility, and where applicable, combination with other datasets, from a technical (database functionality) standpoint. One of the drawbacks in these interdependent relationships is the difficulty in communication and understanding between the different parties. For the relationship and the entire workflow to be successful, correct understanding of the needs and requirements of each role is essential. This is not always straightforward due to differences in terminology definition and mindset in general. A characteristic example is the term “instance”, which can be defined and used differently from data creators, analysts, curators and data managers as can be seen in Table 2, without taking into account the different uses within one specific role. As a result, for the data lifecycle ecosystem to be functional and successful, a moderation between its different parts is needed. In this way, it will be possible to “translate” and communicate between the different parties and guarantee the smooth transfer of data from one role to the other. Furthermore, and potentially most importantly, it will be possible to organise feedback loops to pass evaluations of the usefulness and completeness of the (meta)data coverage and to report issues and errors against the normal data flow to upstream roles for their immediate consideration and fixing and, in more severe cases, rethinking of the metadata concept. This mediation should be done by someone, a “data shepherd”, as the 6th role, combining knowledge and insights on all other roles and requirements. The data shepherd can be described as an enhanced version of a data steward, who not only oversees the data management, handling and quality control processes, but can communicate in a clear and simple language with all parties and resolve any misunderstandings. Data shepherds need to combine experimental, computational and technical background and/or experience and be proficient enough to understand the context in which the different parties express themselves. They will need to lead the data quality control and FAIRness evaluation as well as the continuous optimisation of data workflows, including technical developments to facilitate data curation, annotation, and cleansing.

### 1.3. Quality Management Concepts and Systems—Relevant to NMs and Nanosafety Assessment

Thus far, we have demonstrated the need for implementation of harmonised data management and sharing to solve the complex issues of today in a sustainable and resource-efficient way. Part of this increased drive for data management and sharing is coming from research funders who need to demonstrate a sound return on the investment (RoI). While this is not confined to nanosafety research, it is directly applicable given the increasing influence of nanotechnology, including the upcoming complex and advanced materials to the global economy [43,44].

The EU Open Data Directive released in 2019, extends the Open Research Data policy introduced in the EU research and innovation programme Horizon 2020 and grants full access and reuse of non-sensitive public sector and scientific data. This is part of the European Commission’s Digital Single Market strategy, which is projected to increase the direct economic value from publicly available data, including research knowledge, from €52 billion in 2018 to €194 billion in 2030 [45]. For scientific data, the Open Access and Data Management [46] guidelines require the presence of a detailed data management plan (DMP) on how the data to be collected, processed and/or generated by all H2020 funded projects will be managed and shared. These DMPs need to have detailed provisions on the metadata to be used to increase data FAIRness, based on established standards. If such standards do not exist, the project consortia need to define themselves the types and workflows for the metadata to be created [47]. However, in practice, DMPs have been written, but not all that widely implemented in areas where standards and workflows don’t yet exist, being more of an aspiration than an implementation.

The U.S. EPA and the U.S. Department of Defence (DoD) are both embracing and implementing management tools that fall under the current thinking of continuous process improvement (CPI), quality management systems (QMS), and quality management plans (QMPs). Any organisation or entity conducting environmental or public health programs from which data will inform policy- and decision-making, especially as it applies to regulation, must have a documented quality system (QS) with built in quality management (i.e., the QMS), quality assurance (QA), and quality control (QC). In this context, we first need to consider quality management, which specifies the over-arching organisational management system, which determines and implements policies governing quality, including, but not limited to, strategic planning and coordination, and allocation of resources to operate and manage the QS, i.e., organisational logistics. Second, a system of QA specifies and aligns with an integrated system of management tools and activities that involve the planning, coordination, implementation, documentation, continuous assessment, reporting, and of course quality improvement by a systematic CPI approach. Lastly, QC specifies and describes all the operational procedures, approaches, and activities that are collectively employed to fulfil the already identified and described requirements for data quality, foremost among which, is the value of the metadata.

The QMP documents very carefully that quality system. A QMP helps ensure that generated and analysed data is defensible and trusted by those that are reliant on the accuracy, adequacy, and quality of the data. Simply put, without good sound data, decisions and products are indefensible. In the absence of good data, it remains a challenge to provide a well-informed report that collates various sources of unreliable data, with interpretation of data that might provide inaccurate conclusions or flatly incorrect recommendations, the possibilities of which might include legal and financial liability, loss of organisational and/or personal credibility, and ineffective or worse still fraudulent use of publicly or privately-invested funding and time efforts. Thus, a quality system, of which the QMP is an integral component, is a technical and managerial framework of best practices enabling and strengthening derivation of defensible products and decision-making. The quality system fosters the production of data that is of adequate quality and usability for their stated or intended purpose, and is sufficiently well documented to stand up to the review of a technical system audit or TSA, i.e., quality data has to be “audit-ready.”

A quality system is therefore a system that focusses on quality aspects of a particular research and development program, e.g., environmental health monitoring data. The quality system is documented is documented in elements that comprise the QMP, and the design of that QMP should be specifically aligned to management practices (e.g., good laboratory practices guidelines) and technical procedures (e.g., standard operating procedures or SOPs) that support the program or activity. It is essential to realise that there must be a firm basis for the QMP. Additionally, there must be a quality management system (QMS) already established at the organisational level to document the QMP, and to document existing procedures, develop new procedures where appropriate, and to document any modification or revision of an established QMS. Critically, the QMP, and the QMS that guides the construction of the QMP, is specific and unique to a particular organisation/entity and its component programs and initiatives.

Typically, organisational QMPs define and describe its QA-associated activities aligned to organisational, local and federal policies and procedures, criteria for application of the QMP, specific areas of application (e.g., laboratory-based focused research or field-based studies) and finally, specific roles, responsibilities and authorities. As stated above, it is important to remember that the focus of the QMP is on the quality system. It is a framework and will not provide the fine details that one would otherwise identify with specific work instructions and orders like SOPs, technical directives, guidance documents or quality-assurance project plans (QAPPs). It is critical that a QMP be kept accurate and up-to-date by a process that is coordinated with changes in QA policy, procedures and guidance. Important to this effort, is the need for a succession plan so that any changes to management systems are coordinated by a smooth transition process. For example, should individuals retire, move on to other work functions, and so on, there will be times when the scope of a QMP will change as a result of organisational changes in function, mission focus and responsibility. In those instances, a QMP might require revision. It is critical that a succession plan be in place to permit seamless operational business without any hiatus in oversight and supervisory functions aligned to the QMP and the QMS itself, all of which require clear documentation for record.

QMPs and QMS are the planning and implementation of a set of processes and procedures to help guide, monitor and execute the necessary quality assurance and management of a specific project. Based on continuous monitoring, CPIs allow continuous improvement and optimisation of the established processes. In these cases, DMPs are only part of the entire process for managing data across the lifecycle of obtaining, storing, archiving, updating, and maintaining the integrity of open and closed data streams and warehousing. These process-oriented data management systems share many elements of the product-oriented quality systems associated with the International Organization for Standardization (ISO) standard ISO-9000, especially the product quality portion, ISO-9001. Data management processes are codified, procedures implemented, and performance audited regularly by third parties. Individuals have specific roles and responsibilities including the organisation’s executives. While these are very similar, there are important distinctions. ISO-9000 with more specific quality control targets became the QS 9000 associated with the automobile industry, while the addition of more specific environmental targets leads to the ISO 14,000 series of standards. Good laboratory practice (GLP), is a further refinement adding chain-of-custody logs, sample tampering safeguards and much more frequent calibration testing that culminates in the quality officer attesting to the results under penalty of perjury or other civil action.

**Figure 3:**
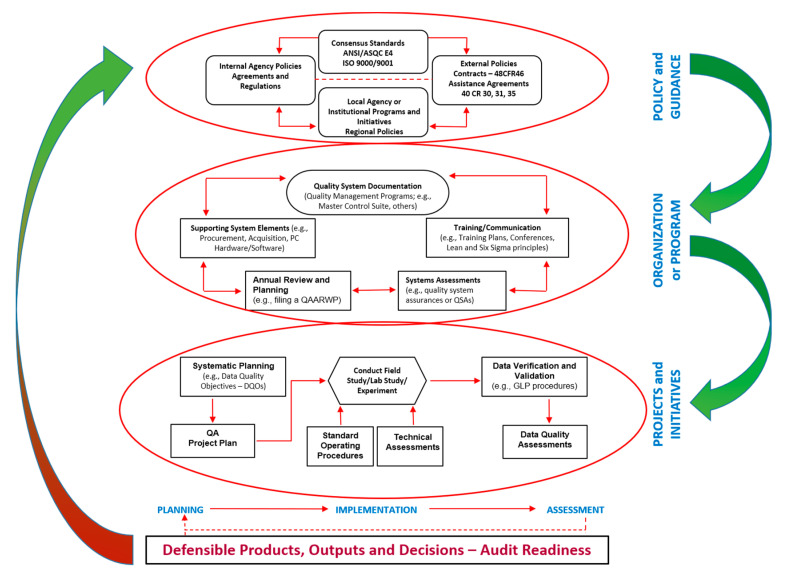
An example of a typical Quality System Components and Tools. The QMS is typically organised across three inter-dependent domains—Policies and Guidance; Organisation of the Program, and Projects, Programs and Initiatives. Adapted and Modified from the U.S. EPA’s technical guidance on Quality Systems and Quality Management [52].

In scientific, including nanosafety, research, such GLP are implemented via laboratory information management systems (LIMS). LIMS were first introduced in scientific research for the management of laboratory samples, analytical workflows and results capturing, Quality Control (QC) and reporting [48,49]. While LIMS are continuously expanded with new functionalities, their rigid workflow-centric structure and IT management makes them appropriate for use in structured (standardised) experiments and sample-focussed workflows and for QC of the produced data [50,51]. However, the collaborative nature of modern scientific projects with partners from academia, small/medium enterprises and large companies, makes the structured, highly focussed and optimised for use inside one company or institution LIMS difficult to implement, harmonise, and interlink due to technical (different incompatible systems) and legal/security issues. In such cases, electronic laboratory notebooks (ELN) are the most appropriate tools for the assembly of diverse data from multiple resources, allowing complex workflow implementation, harmonised data capturing, semantic annotation, mining and exploitation, while allowing adaptation to individual and group-specific ways of working [50,51]. At the same time, ELNs provide a common package of data repositories along with access security, version control, record authentication and automated time stamping which can assist with patenting and protection of intellectual property rights [51]. As shown in Figure 3, LIMS and ELN can be considered as two QMSs that meet different, but complementary requirements, while being data-centric and allowing the capture of complex metadata. However, data capture through LIMS (for GLP compliance) or ELNs (for data organisation, monitoring and sharing) still requires further transfer to appropriate data warehouses for long-term storage. To simplify the process and cut down on the associated working hours and costs, these three should be either combined or linked together to ensure automatic data transfer.

### 1.4. Metadata Types, Schemas, Standards and Semantic Annotation

The ISO/IEC JTC1 SC32 Working Group, which develops international standards for metadata and related technologies proposed that metadata define and describe other data [53]. This means that data can be transformed into metadata in a particular context, but data are not always metadata. There are various types of metadata, which in many cases are context and field specific. In the OECD glossary [54], three main types of descriptive metadata are mentioned:

- Reference metadata: metadata describing the contents and the quality of the statistical data [55].
- Structural metadata: metadata acting as identifiers and descriptors of the data [56], so include ontological aspects such as assay and NM type, instruments etc.
- Statistical metadata: scientific metadata about statistical data [57].

As indicated in Figure 2, there are additional types of metadata, as follows:

- Bibliographic metadata, which include the necessary administrative information for the presented data, i.e., the dataset owner and contact details, the license, the publication status etc.
- Technical metadata: information on the data file types, the size of data, dates of creation and/or modification, types of compression and more, mostly related with databases and interoperability.

In short, metadata offer the context and all necessary explanatory information to make a dataset understandable, reproducible and interoperable. However, they come with the caveat that metadata can be defined in different ways, even within the same field of study. For example, data consumers may request that legal issues be addressed more specifically, such as trade names, use patterns, and quality statements involving chain-of-custody equipment calibration frequency.

**Figure 4:**
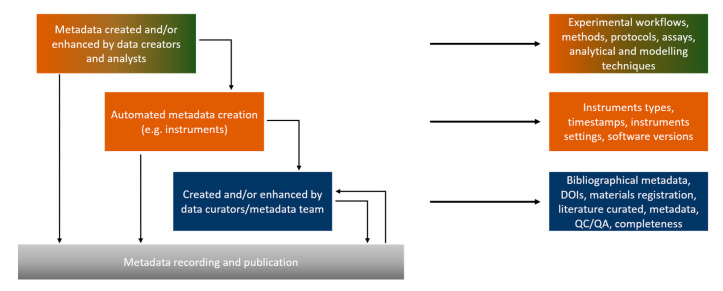
The metadata capture workflow and the respective metadata. Note that the colours in the metadata boxes correspond to the stages of the data life cycle in Figure 2, with orange representing descriptive metadata associated with the planning and acquisition stages, green representing technical metadata from the processing and analysis stages, and navy blue representing bibliographic metadata from the storing and sharing stages.

During experiments, metadata capturing can be divided into three interrelated operational workflow categories (Figure 4). The first includes the metadata created by the data creators (experimentalists, modellers) to design and perform an experimental/modelling workflow. The metadata included in this category are the methods/protocols/assays used to perform an experiment, the analytical and modelling algorithms for data processing, the specific parameters (i.e., the independent variables either set to a constant value during the experiment/modelling and those independent variables allowed to change) used during the experimental and/or computational work performed etc. These metadata are collectively called descriptive metadata (shown in green in Figure 1 and Figure 2). This category interacts continuously with the second, which includes the metadata that are automatically produced and/or originate from the instruments used such as the instrument model and type, the software and software version for data capturing and analysis and the specific standard or user-selected settings (technical metadata, shown in orange in Figure 1 and Figure 2). The instruments’ category feeds directly to the data creators’ metadata, as it can lead to the refinement of workflows and explain deviations from standardised protocols (e.g., ISO, OECD). Both feed into the third category of potential metadata, the bibliographic metadata (shown in navy in Figure 1 and Figure 2), added by the data curators. Data curators are responsible for checking the existing metadata in terms of quality and completeness, requesting further information when needed for completeness and documenting the quality assessment as additional metadata. Curators are also responsible for enriching the produced datasets with bibliographical metadata (e.g., experimentalists contact details, affiliations), assist with acquisition of digital object identifiers (DOI), registration and assignment of unique identifiers to NMs used during the experimental process and semantic annotation using defined ontologies to increase interoperability [58]. Quality and completeness for both the data and metadata will be evaluated either against current standards or most likely against standards/workflows agreed within the project/database they are part of, while, at the same time, extending current standards. Given the continuous evolution of best practices and respective standards, each dataset could include a description of the metadata standards used in accordance with current best practices. This will ensure that the datasets will, in the future, not be flagged of lower quality or lacking necessary metadata.

As noted earlier, experimentalists may themselves act as curators. This creates certain issues when it comes to data completeness, reliability and acceptability relative to scientific disciplines. Those in the physical sciences will likely be less aware of specialised requirements of some data consumers, such as regulatory agencies and the state-of-the-art in some fields (e.g., thermodynamics) may not be as expansive in terms of metadata as the biological sciences. Even within the biological sciences, data users/consumers will differ in terms of their data completeness requirements with some looking to peer review for that purpose, while others will be alert to the additional regulatory metadata requirements [59], such as the Klimisch score [60] when following OECD Test Guidelines for the Testing of Chemicals [61] and good laboratory practice (GLP) [62]. This results in certain databases (e.g., DaNa^2.0^) not incorporating data submissions that do not meet these requirements or in constraining the range of testing considered pertinent to decision making relative to recent advances in the field or acceptable instrumentation or experimental techniques. On the other hand, under the FAIR principles [33] metadata completeness translates into the experiment being reproducible. The metadata in this case can be used to make comparisons, uncover patterns, and develop hypotheses even if they are not regulatory compliant or are mechanistically focussed and thus have broader scope than the purely toxicological studies captured in DaNa^2.0^. Some consensus also exists, within the nanosafety community, that all possible metadata need to be reported for a dataset to be complete, so that data consumers can then decide which metadata, and thus data, are important for them to rely on [63]. This approach of capturing the widest set of data and metadata, offers higher freedom on how to re-use the data and is more supportive of FAIR and data-driven innovation, but puts a large burden on data providers. In the absence of automated data collection tools (e.g., extracting metadata from log files of the experimental equipment), metadata reporting has to be performed manually, often by repeatedly copying and pasting the appropriate fields.

Another barrier to developing a generic metadata schema, which could for example be applied to the entirety of materials science, is terminology use. Slight variations in definitions can lead to significant inconsistencies in semantic use, without taking into account the language barriers in terminology. Similarly, different fields have different requirements in terms of metadata depth, which means that a universal metadata schema would need to be huge and highly complex. Metadata become even more complex the more scientific research advances with movement towards increasingly complex and advanced materials and experimental complexity, i.e., the need to report a material in conjunction with its dynamic environment. This is further enhanced by the disparity in the scientific communities in terms of their interests and resources. While large research institutes and industry have the resources and drive to invest in development and implementation of metadata schemas and standards, SMEs, not-for-profit organisations (e.g., academia), and small research institutes and/or groups may struggle to take part in developing or implementing such practices [64,65]. Some of these barriers could be overcome by simplifying and incorporating such practices early in the experimental design phase supported by software collecting metadata automatically and preparing them in a FAIR way. Automatically created log files from experimental equipment or from modelling and simulation software are one source of automatic metadata creation. Another solution is through the modification of laboratory information management systems (LIMS) and electronic lab notebooks (ELNs) to provide functionality for metadata collection and preparation for upload to data warehouses based on the information the user has provided as described in further detail in Section 1.3.

To overcome these barriers, achieve the desired interoperability, and increase FAIRness, mapping, and communication between the different metadata, schema are needed. This can be achieved using taxonomies or ontologies, which are hierarchically structured domain specific dictionaries [66] that may (ontologies) or may not (taxonomies) contain relationships between the terms at various levels, to supplement the metadata. The terms included in taxonomies/ontologies have specific definitions and are assigned with unique IDs. In this way, it is possible to semantically annotate the metadata with specific terms, which become machine findable and interoperable with similar datasets, and supports indexing of data. To achieve this integration, it is necessary for the different datasets to be annotated using the same or linked (mapped) ontologies to ensure that the various terms are defined in the same way. This requires a complex ontological system, encompassing terms that can describe all parts of a dataset and its accompanying metadata, including the data itself, the units, methods, protocols, statistics, instruments, software used, etc. The most used nano-specific ontologies are the NanoParticle Ontology (NPO) [67] and the eNanoMapper (eNM) [68] ontology. The latter covers various aspects of the biological effects of NMs and is based on full use of terms and branches from other ontologies (e.g., NPO, Ontology of Adverse Events (OAE), Experimental Factor Ontology (EFO), Ontology for Biomedical Investigations (OBI), Chemical Information Ontology (CHEMINF), BioAssay Ontology (BAO)), although losing their inherent hierarchical arrangements, and limiting addition of new terms to those not available in other ontologies. The annotation of NM data with ontological terms increased cross-field data interoperability and resulted in the uptake of semantic annotations in different data management systems [68,69].

However, to reproduce the success seen in the biological sciences and the universal application of ontologies especially in the bioinformatics area [69], there are certain drawbacks to overcome. These emerge from the insufficient coverage of specific areas and inconsistencies in the ontological structure or specific definitions. Similar to the issue of the definition of metadata completeness, this is a result of the extreme interdisciplinary character of nanosafety research, which needs to combine data from very different sources and of multiple types. These require specific terminology and lead to the reuse of the same word in different contexts as showcased for the term “instance” above. Thus, the issue of metadata harmonisation partly shifted to harmonisation of ontologies.

An increasing number of nanosafety projects (e.g., NanoFASE, SmartNanoTox, ASINA) are including an ontology task (supported by NanoCommons Transnational Access) to allow their partners to annotate datasets and in this way increase their interoperability. One successful attempt is the environmentally focussed eNanoGrammar developed by CEINT for the NIKC database. This nano-dictionary was initially loosely based on the eNM ontology, and was created to fit the nature of the data it is trying to accommodate. It includes more terms, specific definitions, and a different relational structure to eNM (which as noted above loses hierarchical structure when importing terms from other ontologies) suitable for capturing the environmental fate, exposure, and effects of NM. ACEnano is continuously collecting terminology used as metadata for physicochemical characterisation data, checks for their availability in existing ontologies or prepares them (including clear, unambiguous definitions) for integration in the most relevant ontology. Such focused activities are good ways to increase coverage and test the ontology structure since they directly target the needs of specific users and might result in separate ontologies targeting specific domains of nanosafety research, e.g., instrument ontologies, methods ontologies etc. However, it is absolutely essential that the developments are synchronised and aligned to avoid incompatibilities and duplication of work. Generalising, one specific ontology to cover all involved aspects might be a way to guarantee this and would take into account that linked databases should be agnostic, with the ontology and its structure being independent from the database-internal data model. Using the examples above, the eNanoGrammar is a dictionary for a specific domain, which could be extended to an ontology inheriting terms from eNM as an upstream, more general ontology. In contrast, ACEnano decided to fit the additional terms into the eNM structure and to request addition of the terms either via NanoCommons extensions of eNM or in one of its downstream ontologies if the terms are more general and useful in other areas. Whatever approach is finally adopted, it will require commitment from the full nanosafety community, along with intensive training to demonstrate the benefits with respect to data FAIRness.

Having introduced the key role of metadata to support interoperability and reuse of nanosafety data, the rest of this paper explores the current state of the art in the implementation of metadata in currently operational nanosafety databases and develops two case studies to demonstrate the value of metadata in facilitating data reuse and sharing across communities. Finally, we provide 8 key recommendations regarding the next steps to facilitate the widespread implementation of metadata reporting, and highlight some key tools and approaches that are facilitating the transition to capturing data and metadata much earlier in the data lifecycle, i.e., at the point of data generation.

## 2. Materials and Methods

### 2.1. Metadata Databases Questionnaire to Build Community-Driven Consensus on Metadata for Nanosafety

The transformation of nanosafety research into a data intensive field and the increased regulatory requirements for nanosafety, has led to a community-wide consensus on the need for data interoperability. Despite the strong community interest and the creation of a number of databases to host nanosafety-related data and dedicated nanoinformatics and e-infrastructure projects to create data platforms, nanosafety data remains fragmented and disparate and is lacking the necessary degree of FAIRness, especially interoperability. As a result, the return on investment (RoI) on publicly funded research is suboptimal, restricting full data exploitation and indeed data driven innovation [70,71]. Many argue that in terms of metadata schemas and/or standards, nanosafety research lags behind other scientific fields such as life sciences and medical research. To get a better picture of the current status, it is important to understand the approach the various data resources take in terms of (meta)data handling and FAIRness. To gather this insight, a questionnaire was shared with nanosafety database owners to check various aspects of FAIRness and metadata existence. Responses (n = 7) were received from the EU, U.S.A., and the Republic of Korea as follows:

- Project databases: NanoCommons Knowledge base, ACEnano, IOM (representing a number of FP7 and H2020 projects including MARINA and PATROLS);
- Institutional or “Centre” databases: Nanomaterial-Biological Interactions (NBI) Knowledge base, Safe and Sustainable Nanotechnology (S^2^NANO), RIVM-ECOTOX and the Center for the Environmental Interaction of Nanomaterials NanoInformatics Knowledge Commons (CEINT-NIKC).

Most of the databases are project- or institute-specific, meaning that the data submission system is only for internal users, with the exception of the NBI, NanoCommons and ACEnano databases. Additionally, for most databases the uploaded data is only made public after a long embargo period decreasing data FAIRness. In the results section, responses to the questionnaire are grouped by topics and specific good-practise examples are presented.

### 2.2. Case Studies to Demonstrate the Value and Relevance of Metadata in Nanosafety Data Harmonisation

The (meta)data gathered by data creators are mainly defined by specific scientific questions. While the captured data can be applicable to various nano-related fields, the metadata and their level of detail may vary depending on the specific research question. The introduction of nanoinformatics requiring large, high-quality datasets makes it obvious that being able to answer the original question, e.g., evaluating the NM activity for a specific toxicology endpoint, is by far not the only possible usage. It is not necessary to look at new and unforeseeable applications because existing predictive models for these endpoints require interoperability of data from multiple resources. To achieve maximum exploitation, it is essential the produced data are usable by diverse disciplines in different contexts. This also requires that some consensus is reached between experts of different backgrounds on the terminology, scientific parameters, media utilised, methods and analytical techniques applied and the minimum information needed to fully describe a specific phenomenon, while achieving data interoperability and reusability.

The behaviour and potential transformations of NMs in different media can lead to different biological and/or environmental effects of interest to a multitude of scientists. The physicochemical characterisation of the starting material or, if this is not possible, detailed information on the sample treatment and fate before the experiment are important metadata to evaluate the influence of such effects on the experimental outcomes of e.g., downstream hazard testing. Characteristic examples of such parameters are NM agglomeration and dissolution, both of which are being studied extensively both experimentally and computationally and have attracted substantial regulatory attention. To demonstrate the different approaches in terms of identifying the minimum information necessary to fully describe a phenomenon and the potential for cross-field consensus, two case studies have been designed and materialised by the authors. The first was a computational workflow studying different scenarios of spherical TiO_2_ and SiO_2_ particle agglomeration in an in vitro biological exposure context to determine delivered dose. This case study identified the minimum set of information required to describe the phenomenon of particle agglomeration and facilitate its application in an experimental context to produce data to validate or disprove the computational conclusions. The second was a questionnaire-based (see Supplementary Information for the full questionnaire) case study targeting NM dissolution experts and seeking their opinion on various aspects of the dissolution phenomenon and studying whether some consensus could be reached on the underlying mechanism, the needed descriptors and the methods of study and analysis.

#### 2.2.1. Minimum Information Reporting on Nanoparticle (NP) Agglomeration as Source for In Vitro Delivered Dose Variations Critical to Human Hazard Assessment

In vitro assays for NP hazard evaluation are still the backbone of early risk screening or detailed investigations of NP-biological interactions on a cellular level. Such assays help to elucidate molecular mechanisms which could result in harmful effects, such as cellular stress responses. These, when not self-limiting, could lead to sustained adverse effects. Cell death by apoptosis or the secretion of immune modulators (cytokines) by professional immune cells as part of a vigorous immune response or inflammation is associated with acute and chronic adverse effects of bio-persistent and inherently toxic NM [72].

In vitro, adherent cells under submerged conditions are the mainstay of cellular assays. The delivered NP dose (DD), defined as the fraction of the administered NP dose coming into close proximity with cells, is a function of particle mass transport over time. Hence, Brownian diffusion and gravitational settling, as principles of particokinetics, are the major determinants of DD and, thus, are important factors to consider when analysing the outcomes of biological endpoints under exposure to non-dissolving NPs [73,74]. NP mass transport by diffusion and gravitational settling depends on NP size and density, and the accurate determination of these properties to characterise the starting material are absolutely essential, but for bioassays are not sufficient to determine DD. Characterisation, providing measured or predicted values for NP size and density, has to include further metadata on how and for what state (initially presented, post-exposure) of the NP they were determined on. Together this information represents the absolute minimum (meta)data, which need to be provided with the dataset describing a biological assay. However, NPs undergo significant and fast transformations in vitro due to agglomeration promoted by molecular entities within the cell culture media. Thus, more information on the fate of the NP needs to be provided as (meta)data to identify the factors influencing the results of the assay. This will allow the data customer to evaluate the interoperability of the offered data with results from other sources, to more accurately estimate changes in size, density and other physicochemical characteristics caused by the environment. Using a specific case study based on an in silico dose modelling approach, we demonstrate how agglomeration of NPs can significantly change the DD and therefore the outcomes of in vitro experiments [73,75,76]. We also propose a minimum set of information that needs to be reported as part of NP agglomeration characterisation for bioassays and how the relevant data and metadata should be documented in published datasets to fulfil the requirements of the scientific FAIR principles described in Section 1.1. To our knowledge, agglomeration metadata accompanying NP in vitro bioassay datasets are extremely rare despite being essential in order to determine the consequences of the DD as demonstrated below.

##### Objectives of This Case Study

- To determine the impact of a few different agglomeration scenarios (primary particle vs. well-defined agglomerate vs. three different mixtures thereof) of two types of NPs (TiO_2_ and SiO_2_) on biological in vitro endpoints.
- To collect (meta)data regarding particle agglomeration, which are relevant for in vitro experimentation using adherent cellular models.
- To define a minimal set of information (data and metadata) most relevant for NP agglomeration to facilitate interpretation of DD in in vitro bioassays.

##### In Silico Modelling of NP Agglomeration

To conduct the case study, two types of model primary NPs, SiO_2_ (density 2.2 g/cm^3^) and TiO_2_ (density: 4.24 g/cm^3^), both with a size of 50 +/− 0 nm and spherical shape, and agglomerates (250 +/− 0 nm, spherical shape) thereof were established. Three different mixtures of primary NPs and agglomerates were defined. Hence, for each particle type the deposition of NPs, in five different scenarios, was simulated: primary particles only, 80% primary particles, 50% primary particles, 20% primary particles and particle agglomerates only. DD prediction within 24 h at an administered dose of 10 µg/mL was performed for each particle type and mixture by in vitro submerged simulation using the in vitro sedimentation, diffusion, and dosimetry (ISDD) model (current version Nov 2019) [77]. To calculate the effective density of agglomerates, a packing factor of 0.637 (default value of ISDD) and H_2_O as medium were used. The detailed parameters are given in Section 3.2.1.

#### 2.2.2. NMs Dissolution: Achieving Consensus on Terminology and Metadata Usage

Achieving greater uniformity in metadata usage, whether pursued within just the multidisciplinary nanosafety community or more broadly to all disciplines through the FAIR initiative will alter everyday practice and the tools, e.g., LIMS, ELNs, etc., used. In order to gauge the implications, we surveyed colleagues whose work had involved the measurement or interpretation of dissolution data. Dissolution was chosen as the focal point as it had been proposed as a pilot project in the EU-US Nanoinformatics Roadmap 2030 [40] and has gained increased regulatory attention. Materials that are highly soluble or dissolve quickly are not biopersistent and the toxicological testing of the dissolution products, not the NM, may suffice. Lastly, issues surrounding confidential business information regarding dissolution are unlikely, allowing for extensive collaboration among industry, government and academia.

Yet, there are issues to consider. Dissolution and solubility are distinct concepts that have been used interchangeably in the nanosafety literature. For example, the EU’s Scientific Committee on Consumer Safety has issued an opinion [78] on silica solubility in response to an industry proposal that there is a threshold solubility [79] above which one could assume that dissolution is complete [80]. There are similar issues in the nanomedicine field where the deliberate release of a therapeutic agent through dissolution is described as analogous to the uncontrolled release of a toxicant shed by a particle, though these rates and volumes of effect can be quite different.

A survey tool was generated (see Table 3 for an overview of the suggested metadata and Supplementary Information for the full questionnaire and a summary of the results) utilising a flow sheet concept comprising: terms and definitions; a recommended unit of measurement; a catalogue of competing reactions and induction time effects that should be considered; current standardised media and methodologies; and the models used for data interpretation. The questionnaire was sent to approximately 70 colleagues globally who had some experience in interpreting dissolution data for either nanosafety or nano medicine purposes. Eighteen of those queried responded.

## 3. Results

### 3.1. Community-Driven Consensus on Metadata for Nanosafety

#### 3.1.1. Data Coverage

Most of the nanosafety databases include both experimental and literature curated data. Of the 7, ACEnano and the IOM contain experimental data only. ACEnano has the additional feature that it only reports physicochemical characterisation. It is specifically optimised for new, emerging methodologies without standard operating procedures (SOPs), as well as interlaboratory comparison experiments (round-robins). Both ACEnano and IOM demand detailed coverage of the protocols used to allow comparison of the produced results (see below). The NBI and S^2^Nano databases focus on the biological activity and toxic potential of NM, while RIVM-ECOTOX and CEINT-NIKC cover ecotoxicology data. IOM contains data spanning a wide spectrum of nanosafety research, as it is populated with data from various EU funded projects, like MARINA [81], SUN [82], NanoSolutions [83], PATROLS [84], Gracious [85], and more. Similarly, the NanoCommons Knowledge Base is built to accommodate a wide range of nano-related data and is currently hosting data from NanoMILE [86] (bio-nano interactions, physicochemical characterisation, toxicity and ecotoxicity), NanoFASE [87] (environmental fate, exposure and hazard), NanoFARM [88], SmartNanoTox [89], and other projects, as well as computational and literature data.

IOM is also on course to start accommodating simulation data, through its partnership with the NanoInformaTIX project, while the NBI, NanoCommons and S^2^Nano, which already accommodate such data. Computational and simulation data are becoming more and more prominent, due to the continuous evolution and increase of nanoinformatics, as expressed through the EU-US NanoInformatics Roadmap 2030 [40]. This results in an increased demand for data management and sharing from such projects.

#### 3.1.2. Metadata and Data Templates

The data contained within the reviewed databases is curated under specific guidelines in 4 of the cases, although some forms of templates are used in most cases (mainly ISA-TAB-Nano based templates [31], see below). However, the different approaches taken show that there is still a large uncertainty regarding the best way to present metadata with respect to its content/completeness. For example, data templates created by one project are adopted by other projects only to a very limited extent and their use almost always requires adaptations and customisations. Often such templates are focusing on technical details such as encoding language (HTML or XML) or the file format (CSV vs. JSON vs. MS Excel), as well as its associated schema (defined below), which might easily become outdated. The assumption is that data are transferred from the creator to the data manager and finally to the data customer using the same format. Instead, the current field-specific nature of metadata combined with the need for data to be interoperable should lead to the establishment of metadata requirements aiming to facilitate cross-field dataset understanding for both humans and machines [90]. In terms of reporting, metadata requirements are considered essential in the majority of the databases (ACEnano, NanoCommons, IOM, CEINT-NIKC). These databases include and/or request all types of relevant metadata (bibliographical, descriptive, technical, as per Figure 2). The rest consider metadata desirable and include either bibliographical and technical (RIVM-ECOTOX) or descriptive (NBI) metadata. To further guide the implementation of metadata, a short summary of template development in general followed by nano-specific definitions is provided.

For reporting scientific research, schema focusing on publishing data in journals and databases often lack the ability to accurately define the meaning of a specific metadata or data element [54]. The field specificity of metadata and the need for interoperability, has led to the establishment of metadata requirements to be addressed during data collection to facilitate a common understanding of the meaning and representation of the data and make them both human and machine readable [48]. Metadata requirements can be either generic (e.g., Dublin Core [91], DOI [92]) or field specific (e.g., ISO 19,115 for geographic data and Darwin Core for biodiversity informatics [93]). The latter requires community consensus and the development of specific guidelines on the types of metadata to be captured and how to store it, along with clearly defined terms and is called a metadata schema. Standardised schemas, which are developed, maintained and improved by specific organisations constitute metadata standards (e.g., ISO, ASTM). The metadata schema can be supplemented with specific rules regarding the format, content and allowed values for all items. Metadata schema aim to harmonise and promote compatibility and interoperability for the data produced within a specific field, which is more easily achieved observational data [94], than for a conceptual model. The common practice is to define a minimum amount of information needed compared to complete metadata reporting, but still conform to quality standards. Examples of such practices, directly or indirectly related to nanosafety research, are the Minimum Information for Biological and Biomedical Investigations (MIBBI) [95], Minimum Information Reporting in Bio–nano Experimental Literature (MIRIBEL) [96], and Minimum information about Nanomaterial Biocorona Experiments (MINBE) [97].

To make the metadata machine readable, achieve harmonisation and increase findability, metadata schemas can be encoded using HTML or XML languages [94]. One of the first metadata schemas, and subsequently standards, to be developed in 1987 was the Directory Interchange Format (DIF), which was initially built for reporting geospatial data [98]. While DIF has a flexible structure for accommodating growing metadata requirements, the launch of ISO 19115/TC211, and other similar metadata standards, led to DIF being redesigned to achieve maximum interoperability. This demonstrates another risk, arising from the existence of multiple metadata standards within one field, which can lead to substantial barriers for data sharing, interoperability and reusability [64]. This is further enhanced by efforts towards cross-field data sharing via informatics. On the other hand, the use of generic scientific metadata standards like the Core Scientific Metadata Model (CSMD-CCLRC) and the ISA-TAB standards can be constrained by fear related to the loss of field-specific data and historical community practices and workflows, as reported by Willis et al. (2012) [64], although many metadata-driven goals are independent of scientific discipline.

The ISA-TAB-Nano extension [31] of the ISA-TAB standard was the first attempt to create a structured metadata schema focusing on nanomedicine and include the option to semantically annotate the reported data. This was further adopted and modified by the NANoREG and subsequently eNanoMapper and Nanoreg2 projects. ISA-TAB-Nano is a complex CSV-type file structure, which allows the capture of complex experiments, but requires different files to report different types of experiments (e.g., physicochemical characterisation, in vitro or in vivo assays) and is not designed to account for environmental fate and behaviour studies of dynamic entities as NMs. Another reporting format was defined by the CODATA-VAMAS working group in 2015 (and revised in 2016), the Uniform Description System on the Nanoscale (UDS) [99]. UDS focussed on describing the synthesis as well as physicochemical and structural characterisation of NMs and did not account for complex experiments, their surrounding environment, or the use of computational descriptors and nanoinformatics approaches that are becoming increasingly prominent. ASTM Committee E56 on Nanotechnology has created three Standard Guides or metadata related to the description of nanomaterials [100,101,102].

A recent attempt to develop a data capture template, and not a distinct metadata schema, which incorporates the reporting of complex experimental data along with detailed respective metadata, is offered by the CEINT-NIKC [103]. The NIKC template was developed for the joint reporting of both the NM characteristics and its surrounding environment, while incorporating the different metadata types along with detailed protocols/assays and instruments/consumables used. The CEINT-NIKC template was modified (simplified and streamlined via the H2020 NanoCommons infrastructure project) for use by the H2020 project NanoFASE, in an attempt to capture complex mesocosm experimental data. The main benefit of NIKC is that it can accommodate all types of nanosafety experiments, including simple computational workflows, although it is limited by the lack of a nano-wide ontology for the annotation of terms, an issue being addressed currently within NanoCommons.

#### 3.1.3. New Challenges Arising from Nanoinformatics

**Figure 5:**
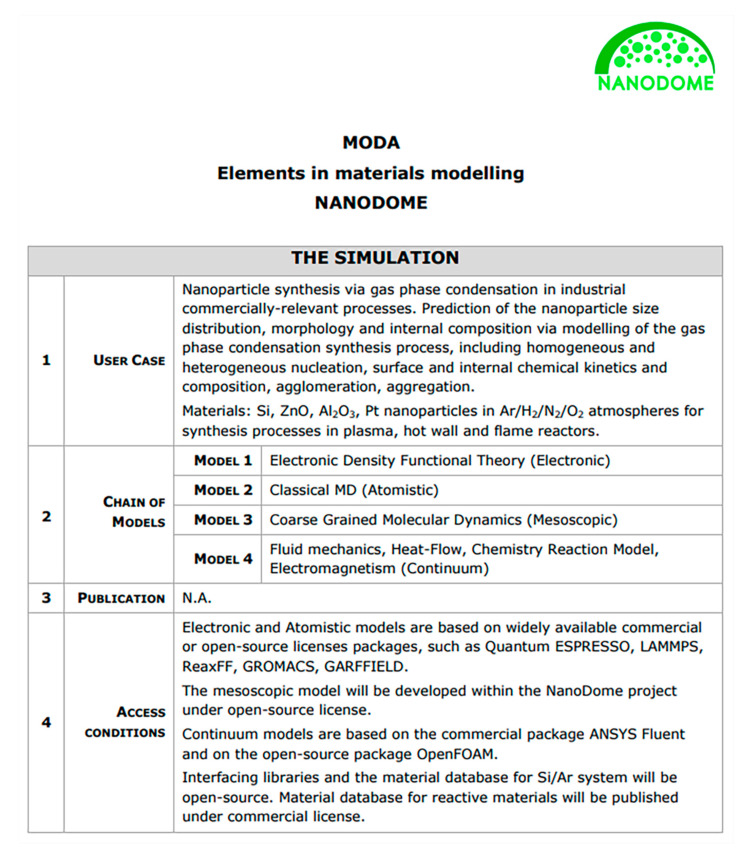
An example MODA template for nanoparticle synthesis and computational prediction of the size and size distribution.

The issue of the data format required for computational workflows, and its differentiation from currently used formats, has been stressed in the EU-US NanoInformatics Roadmap 2030 [40] and is now beginning to be addressed in conjunction with the development of computational specific templates. Examples include nano-specific computational templates based on the Minimum Information About a Simulation Experiment (MIASE) [104] guidelines, the Modelling Data (MODA) templates [105] and nanoQSAR reporting formats, being expanded by NanoCommons, SmartNanoTox and NanoSolveIT. Furthermore, the NanoSolveIT project is using the Enalos Cloud Platform to develop a dedicated database for computational ready-to-use data. These attempts take into account the increasing number of computational descriptors at various levels and the specific needs for the reporting of in silico workflows. The MIASE guidelines state that a model must be identified, accessible and the steps of the simulation including any post-processing steps must be fully described, with detailed user guidance, and the output or dependency of results should be reported. MIASE built on its precursor Minimum Information Required in the Annotation of Models (MIRIAM) [106] guidelines, which aimed to promote better reproduction of simulation results. MODA templates, now in their third version [107], provide a structure where researchers provide the metadata necessary to describe, reproduce and curate multiscale workflows, allowing interfacing with other models. Several nano-related MODAs have been made available [105] covering diverse areas including modelling the properties of complex nanomaterial structures produced by gas-phase synthesis (Figure 5), and for the fabrication of nano-porous carbon. The MODA template allows researchers freedom to provide the information required, using fixed terms or free text entries, which comes at the cost of impeding machine readability. Clearly, efforts in this area will benefit from the development of the European Materials & Modelling Ontology (now in its first, alpha release [108]) that will allow annotation with corresponding ontology terms.

Another important modelling approach is the highly versatile nanoQSAR modelling [109]. Such models have wide applicability, and have been used, among others, to predict the cytotoxicity [110,111,112,113,114,115,116] of NMs in human and animal cell lines and the thermal conductivity of nanofluids [117,118]. Besides QSAR [110,111,113,115,116], QFAR (quantitative feature activity relationship) [112] and QSTR (Quantitative structure toxicity relationship) [114] modelling have also been employed. Several studies have used Simplified Molecular-Input Line-Entry System (SMILES) and quasi-SMILES approaches for model development [110,111,112,113,115,117,118], or enriched the dataset with atomistic computation and molecular descriptors to help decode the cytotoxicity mechanism of metal oxide NMs [116]. To accommodate this model versatility and assist with reproducibility, the OECD recommends that QSAR models are accompanied by a QMRF report (QSAR Model Reporting Format) [109], a harmonised template for reporting the key features of QSAR models, including validation information, structured according to the OECD principles for the validation of QSAR models [25]. The OECD principles, embedded in the QMRF report, require the modellers to provide the necessary information so that the modelling workflow is described using in a reliable and transparent way to assist regulatory authorities in the acceptance of QSARs as non-testing ATS [25]. These principles include a defined endpoint; an unambiguous algorithm; a defined domain of applicability; appropriate measures of goodness-of-fit, robustness and predictivity; and a mechanistic interpretation, if possible.

**Figure 6:**
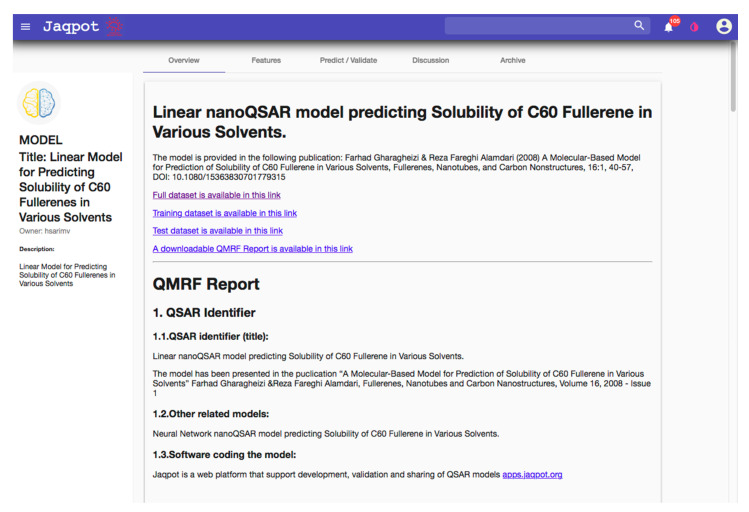
Metadata report (QMRF) of a QSAR model in Jaqpot 5 (NanoCommons platform).

The reproducibility and transparency of nanoQSAR modelling requires user autonomy, ability to share models with the community for predictions and testing, accompanied by their QMRF report, and a space to receive feedback and reply. A platform that provides teose functionalities is Jaqpot 5, part of the NanoCommons nanosafety research infrastructure, which allows users to upload their models and make them available as web services so others can run, evaluate and comment on their performance. An example of a QSAR model with a QMRF report available on the Jaqpot 5 platform is shown in Figure 6. QMRF reports can be created either using the QMRF editor provided by JRC [119] or by editing a QMRF markdown template [120]. Additionally, QPRF (QSAR Prediction Reporting Format) reports [109] should accompany property predictions, describing the evaluation of a specific model for a specific substance. Just like experimental results, modelling results can exhibit variations, which need to be understood, explained and reproduced in order for the results to gain the trust of users.

The above efforts provide guidance for researchers to provide information to allow visibility of a model and understanding of its results. For NM models, the accompanying metadata should also describe the materials involved fully (composition, structure, history, protocols) and the precise definition of the modelled endpoint, so that differences in values can be effectively interrogated.

The development and encoding of (meta)data templates are highly significant when it comes to data discoverability and reusability for meta-model creation. Meta-models for intrinsic and extrinsic NM properties can facilitate the development of robust nanoinformatics predictive models assisted by machine learning (ML) and artificial intelligence (AI) techniques and need a large amount of data to be effective. This can be done with the identification of the relationships between the structure of NMs (represented by descriptors) and computed or simulated intrinsic and extrinsic NMs properties. The result will be the development of predictive “meta-models” for the estimation of computationally demanding atomistic, quantum-mechanical (QM) and molecular dynamics (MD) simulations of NMs. In subsequent steps, these meta-models can be used for the development of cost effective nanoinformatics tools and models based on AI for the prediction of crucial NMs functionalities and adverse effects and subsequently safer-by-design approaches. A characteristic example is the further exploitation of transmission electron microscopy (TEM) images, which are obtained to determine the size and the shape of NMs and other morphological characteristics, which can then be integrated into modelling workflows to generate additional computational descriptors in order to identify the best correlation with NM behaviour and biological effects. the NanoXtract tool [121], developed under the NanoCommons project, has been used to extract a range of different variables from TEM images, and the image nano-descriptors were further exploited to derive a validated predictive model to estimate zeta potential of NMs. Using ML approaches, images of Daphnia magna exposed to a range of ENM were utilised to develop a model for prediction of ENM-induced ageing and toxicity [122].

From the responses submitted to our metadata questionnaire, the NanoCommons, NBI, CEINT-NIKC and IOM databases consider images to be a type of metadata. Based on the IOM response, besides information on where and when they were captured, images can contain information about the image itself in image-file-inherent metadata (if preserved by the technology/workflow). Such information can assist with image handling during their processing and analysis for the development of deep learning models. Additionally, the file format selected should be open in order to promote accessibility and communication of results amongst users of different instruments or software; a tool that helps advance this is Bio-Formats [123], a standardised library that enables reading from many life sciences proprietary file formats and outputs to open ones, while transferring the image acquisition metadata into a common open data model.

Several studies [116,124,125,126,127,128,129,130] have demonstrated the benefits of meta-analysis through data combination and reusability for assessing NM toxicity. A characteristic example is that of Labouta et al. (2019), in which the meta-analysis of 93 peer-reviewed papers, corresponding to over 3000 data points on the cytotoxicity of organic and inorganic NMs, established decision trees combined with feature selection methods to reveal hidden relationships [130]. Most of the existing meta-analyses studies [116,124,125,127,128,129,130] have stressed the significance and/or effects of missing metadata information on the produced results reliability (e.g., explaining outliers or low accuracy) or have explicitly included them as experimental parameters (e.g., assay type) to study their significance and correlation with observed toxicity. A meta-analysis by Wilhelm et al. (2016) on the efficiency of delivery of organic and inorganic NM to tumour tissues, used bibliographical (e.g., document type, keywords, language) and descriptive metadata (e.g., experimental replicates) to limit the studies used (from over 40,000) to 117 of the “highest quality” [126]. No further metadata usage is reported for the analysis, but the favourable results led to the development of the Cancer Nanomedicine Repository (CNR), a database for the submission of relevant data [131]. CNR is an open database where experts can submit their datasets, but no specific metadata are requested with the exception of the DOI linked to the published research paper and the method used to quantify the concentration of the NM in the tumour.

Gernand and Casman (2014) performed a meta-analysis on a collection of 136 types of CNTs from 17 publications and used regression trees to correlate the pulmonary toxicity of CNTs based on 41 controlling factors (including intrinsic and extrinsic independent variables) including impurities, CNT geometry/dimensions and exposure characteristics [125]. They acknowledged that the lack of standardised characterisation protocols and reporting was a limiting factor in the model success and accuracy. To overcome these limitations, the dataset was expanded to include both NMs properties and experimental conditions, which were treated as independent variables to assist with data interoperability and analysis. The exposure mode was found, for the specific experimental conditions, to have minimal effect (0.001–0.5%) on the overall dataset variance reduction. On the other hand, a number of studies [116,124,127,128,130] found a significant correlation between the cytotoxicity assay type and cell viability, while one [129] found a significant correlation between the NM dispersion protocols and cytotoxicity. All stressed the significance of protocol standardisation to reduce inconsistencies between studies, with Labouta et al. (2019) limiting the studies used for analysis to those involving commonly used cell viability/cytotoxicity assays in order to overcome the challenge of integrating heterogeneous cytotoxicity data [130].

**Figure 7:**
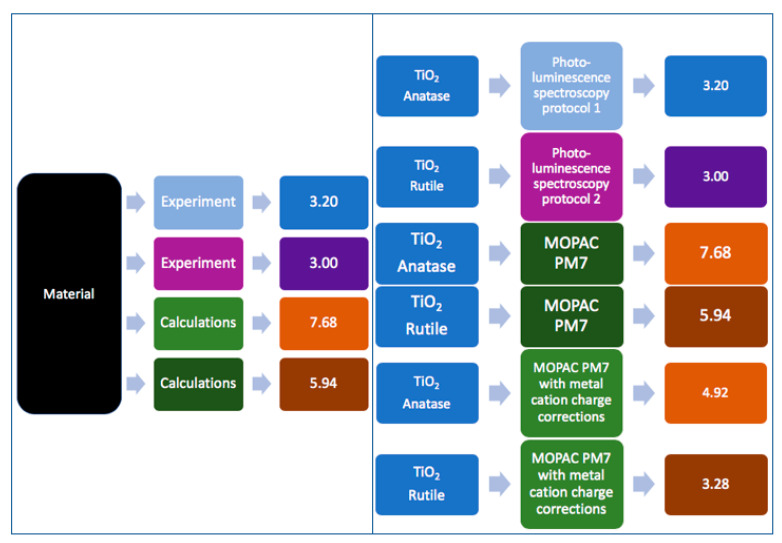
Integrating data points without paying attention to the metadata (left), provides little information, while a careful examination of metadata reveals the independent variables influencing experimental and computational results, allowing insights into how they came to be and a more informed choice on which values to accept and use.

Another issue that arises when combining datasets from different studies is data gaps and how to fill them. Bilal et al. (2019) commented on the complexity of data originating from different experimental platforms/settings and the need for advanced data mining tools and extraction techniques to acquire as much information as possible from integrative analysis of the body of evidence [124] as demonstrated graphically in Figure 7. Combining data without paying attention to the production source may lead to increased uncertainty, as data may have been produced using various experimental or computational workflows. Similarly, Ha et al. (2018) demonstrated the benefits of data gap filling using techniques like supplier specifications, references using the same NM or computational estimation from other physicochemical properties in meta-analysis and provided a grading system that evaluated the dataset quality based on each parameter’s metadata (i.e., origin of measurement/calculation) [128].

#### 3.1.4. Data Management, FAIRification and Reusability

One of the key aspects of databases is, or rather should be, the establishment of data management processes that allow further exploitation of the hosted data. Any exploitation outside the original data scope and ownership should add value to the data itself, and for the data creator/owner through proper attribution/citation of the reused work and the database hosting the data. Implementation of a clear ownership and licensing system, in combination with the assignment of unique identifiers (e.g., database specific, DOI) is central to achieving this, and is best achieved through use of data management plans (DMP).

Of the database responses to our metadata questionnaire, three of the EU-based databases, namely the ACEnano, NanoCommons, and IOM DMPs, in the case of IOM are project specific. In most cases, these are confidential and available only to consortia members, with the exception of NanoCommons which has published and is periodically updating its DMP [132] as part of its efforts to enhance data management and re-usability across the nanosafety community. All databases that responded, provide fully or partly open access data. The latter refers to embargoed data that is available only to data owners and/or relevant partners or following individual agreements for a fixed period. More data are becoming publicly available as the embargo period ends or as relevant datasets are being published. In terms of referencing/attribution of the existing data, the NanoCommons, ACEnano and IOM databases offer either an internal unique identifier and/or DOI assignment. These databases, including CEINT-NIKC and S^2^Nano, offer a licensing system for all data they host, especially if publicly available, and ownership remains with the data creators/providers. In this way, the appropriate attribution is facilitated, which is expected or requested in all databases. As commented by the IOM submitters, there is the belief that specific processes to monitor data reuse are not sufficiently, if at all, implemented in current databases, either procedurally or technically. This is further enhanced by the question of long-term sustainability of these databases and who will ensure and enforce proper reuse, taking into account the current Open Data requirements of the European Commission.

In terms of FAIR metrics and FAIRification, we quickly present the current status from the reviewed databases, specifically on the technical FAIR principles according to the classification above. The NanoCommons, IOM and NBI databases claim to provide FAIR data, although this is mainly true for newly curated data in the case of the IOM database. Persistent unique identifiers, defined access protocols in the form of download formats and licenses have been discussed above. All 7 databases that responded provide search features but registering or indexing for a unique identifier in an external searchable resource is often still left to the data provider. Such a service is Zenodo, which offers the potential to upload content (e.g., publications, reports datasets) under different licenses, DOI them, and make them FAIR.

#### 3.1.5. Metadata on Test Methods and Protocols

While the attempts presented so far focussed on the reporting of data and the relevant metadata, they did not specifically stress the need for reporting of detailed experimental protocols and assays, or indeed computational workflows, to facilitate data reproducibility testing. An important attempt towards that path was from the NFFA project [133]. NFFA developed and proposed a complex metadata schema based on the UDS system, combined with other existing schema like vCard, FOAF, CSMD, CERIF, and more, and aimed to report all possible metadata, including used instruments and protocols/assays. Unfortunately to the authors’ knowledge, the proposed standard is limited to applicants using the NFFA infrastructure and no detailed public guidelines currently exist on the use and filling of the schema independently.

Based on the questionnaire responses, the IOM, ACEnano, NanoCommons, and NIKC link to specific protocols used to produce the data to the dataset. However, ACEnano is going one step beyond the other databases by making the protocols a central step of the data upload process and integrating information extracted from the protocols directly as metadata. The starting point of this unique approach is the ACEnano protocol module, using highly structured questionnaires to document all experimental steps and specific settings in an annotated and computer-readable way. Three different types of protocols are supported: sample preparation, measurement and data analysis. For example, the user provides detailed information on the storage, dilution, heating or sonication processes during the sample preparation and details on the equipment starting from type and producer of complex machinery down to pipets in the measurement protocols to document even small variations performed for specific samples. All this information is annotated with ontology terms supported by an ontology lookup service [134]. For the data upload, defined workflows are provided in the data module, in which information on the data provider and the specific NM sample under investigation is collected, then one of each kind of protocols is linked and finally the datafiles are uploaded. The data annotated with the metadata from the protocols and the additional information provided within the data upload workflow can then be searched, browsed and accessed via the ACEnano warehouse user interface or via application programming interfaces (APIs) for their direct integration into downstream analysis and modelling workflows.

A similar approach has been established by the CEINT-NIKC database and the respective data templates. NIKC has linked all data submitted to the database to both the methods and instruments used to produce the data. This approach was initially employed to allow interoperability of complex ecotoxicological experimental data (mesocosm experiments) through the collaboration between CEINT and the NanoFASE and Serenade projects. NanoCommons project adopted this approach and included analytical metadata on the methods and instruments used to all templates for data curation and uploading. Furthermore, NanoCommons in collaboration with NanoFASE expanded the NIKC templates to accommodate automatic semantic annotation of the datasets using ontological terms, which are mapped to those used from the NIKC’s nano-dictionary and to the eNM and associated ontologies.

#### 3.1.6. Quality Assurance and Quality Control

Quality assurance and control (QA/QC) of data are key for increasing data reusability and providing confidence to data customers and needs to be addressed at the community level. Currently, the nanosafety field is lagging behind life and medical sciences in reporting the metadata needed for quality evaluation, although substantial attempts are currently underway to improve this. Even published datasets, in several cases, do not disclose the full protocols (or algorithms, source codes for simulation data) used, or reference other publications that are difficult/time consuming to access or in some cases irrelevant to the current publication.

The issue with the quality of both the data and metadata has been discussed in the previous paper of this series by Marchese et al. [28]. Currently, most databases do not follow an established quality management system, with only a few using the ISO-9001 for data quality and/or ISO-27001 for data security. This either moves the workload for manual QC of the datasets to the data curators, as is the case with CEINT-NIKC, or if this is not possible it is left to the submitter/uploader to QC the dataset individually without clear guidelines and requirements, which is especially problematic when it comes to metadata that are more difficult to retrieve post-experimental. This is further enhanced by lack of knowledge or incorrect (old-fashioned) mindsets regarding the need for detailed DMPs at the very beginning of projects spanning their entire lifecycle, and the allocation of substantial QA/QC processes for metadata testing and gap filling and the current lack of available tools to facilitate this. It is important to note that the presence of the ISO-9001 system in a database does not mean that the submitted data are of the required quality and uploading does not improve the dataset quality. On the other hand, if ISO-9001 compliant quality measures are in place during uploading, prompting the user to submit information on the processes/workflows and standards used for data creation, they will force the submitting party to add information that may have been initially omitted.

In terms of metadata and semantic annotation, most of the databases that responded to the questionnaire require the submission of all types of metadata, although a QA/AC tool for metadata is currently lacking from all except the S^2^Nano database. S^2^Nano has implemented scoring systems like the INFO score from bibliographic metadata (journal name, journal information, etc.), PChem and Tox scores from technical metadata (the methods and protocols used to produce the data, instrument details and settings). Generally, there was consensus among the database owners that the most common challenge is to gather the necessary metadata, especially in a standardised format further emphasising the need for a metadata standard for nanosafety research. At the same time, there needs to be a balance between strict standardisation and allowing users the necessary freedom to fully represent their data. This is why some fields are not mandatory, which results in databases relying on the willingness of users to fill in all the required information and the increased difficulty to create inter-dependencies between metadata information for complex workflows.

Most of the surveyed databases have some form of metadata functionality to promote data interoperability, combination and reusability via higher quality and bigger datasets. Ontologically, most databases (4/6) use either specific ontologies (e.g., eNM, NPO) or one of the available lookup services (e.g., EMBL-EBI, NanoCommons). The use of lookup services instead of specific ontologies results from the fact that terms may be missing specific definitions when added into or inherited between different ontologies. Another issue is the context-specific definition of terms and reuse. In such cases, the correct term may be identified in various ontologies, but the definition may change based on field-specific usage. This creates significant uncertainties pertaining to the applicability of a specific term for the annotation of specific (meta)data and is one of the main issues data curators and database owners face. However, some of the difficulties with the use of more general ontological services is the uncertainty of which term to use when that term is listed in several repositories. Until this is solved (e.g., by the ontology development activities shortly introduced above and the identification of synonyms), manual mapping between the different ontologies will be required for databases to be able to communicate between each other and the data to be interoperable.

#### 3.1.7. Metadata Awareness and Training

It is not by chance that all responders named metadata acquisition as the main challenge they face. Metadata completeness and quality are also mentioned as key obstacles. In many cases, metadata are confined by the funding available and the needs of individual researchers to publish their data. This is especially true for NM characterisation, where no publications standards exist, leading to huge discrepancies in reporting. Lack of sufficient metadata inhibits database managers to implement tools that would help enhance, enrich, promote and increase the interoperability and value of the datasets. Based on the survey responses, tools for (meta)data handling, processing, analysis, and semantic annotation are needed.

To promote metadata capturing and further exploitation, the community needs to be informed and educated, within clearly defined guidelines, on data quality, interoperability, reusability and their benefits for the wider community and at an individual level. Consensus is needed on this guidance with the FAIR principles, both scientific and technical, being the best candidates to date to show the way forward. The introduction of FAIR metrics and FAIR scores for individual datasets, as well as relevant databases, is expected to lead to substantial increase in data quality and interoperability. In fact, all database respondents, who did not have an established FAIR data system, were interested to learn more and to implement such a system. To achieve this, a realistic approach for metadata capturing is needed.

### 3.2. Case Studies to Demonstrate the Value and Relevance of Metadata in Nanosafety Data Harmonisation

#### 3.2.1. Essential Information Reporting on NP Agglomeration as a Source of In Vitro Delivered Dose Variations Critical to Human Hazard Assessment

##### Impact of Particle Agglomeration on the Time Scales of Biologic Responses

**Figure 8:**
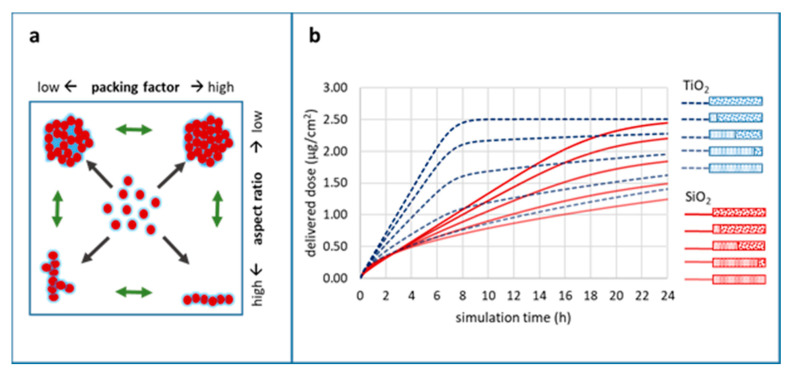
Agglomeration and its impact on the delivered NP dose in in vitro experiments using adherent cells. (**a**) Depending on NP characteristics and environmental conditions, primary particles may build agglomerates of different types (black arrows). This results in changes of effective density (packing factor) and shape (aspect ratio) of the newly formed agglomerates. Effective density is determined by the accumulation of intra-agglomerate fluid. Unstable agglomerates may change packing factor and shape with time (green arrows). (**b**) Different agglomeration types lead to alterations in particle mass transfer (particokinetics) and result in different delivered doses in in vitro experiments. Delivered dose prediction for two different particle types and five different mixtures of primary particles and agglomerates was performed by the ISDD in silico method simulating an in vitro submerged model. The following parameters were used for simulation (for details see Table 4): particle types: TiO_2_ (blue), SiO_2_ (red); primary particle diameter & shape: 50 nm, spherical; agglomerate diameter & shape: 250 nm, spherical; particle mixtures (agglomerate (%): primary particle (%)): 
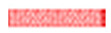
 100:0, 
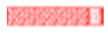
 80:20, 
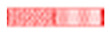
 50:50, 
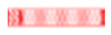
 20:80, 
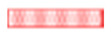
 0:100; medium: water; dish depth: 0.25 cm; administered dose: 10 µg/mL.

In silico DD prediction modelling for 24 h, which is a common incubation time for in vitro experiments, unveils that NP deposition is not very sensitive to differences in particle density (TiO_2_ vs. SiO_2_), but it is very sensitive to particle agglomeration promoted by molecular entities within the cell culture media (Figure 8a). For the agglomerates there is a near to complete conversion of administered dose to DD at 24 h (Figure 8b). This is not seen for primary particles, where the DD half the administered dose. The situation is quite different at 4 h, a typical time point for mRNA expression analysis, because neither of the NP types or agglomerate mixtures reaches particle exhaustion, the point where particle deposition ceases. For SiO_2_ NPs, DD is not significantly different between primary particles and agglomerates, in contrast to TiO_2_, where agglomeration strongly increases the DD, as shown in Figure 8b.

**Figure 9:**
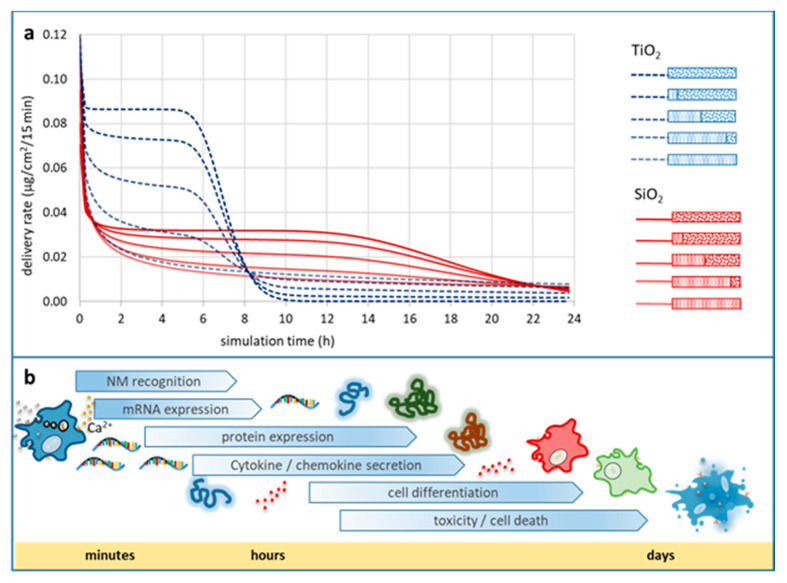
Impact of NP agglomeration on delivery rate in relation to the kinetics of biological responses (endpoints) typically monitored by in vitro experimentation using adherent cells. (**a**) Dependence of the delivery rate on NP/agglomerate density depicted for different mixtures. All parameters unchanged from Figure 8. (**b**) Time spans of typical biological readouts.

In general, higher primary particle density leads to increased delivery rate (DD/minute) due to gravitational settling. Much more importantly, our data suggest that agglomeration acts as a multiplier of the early-phase delivery rate (Figure 9a). Hence, agglomeration may induce early overload effects in vitro, which do not anymore reflect the intended dose to assess in vivo exposure scenarios, although the in vivo tissue DD and the in vitro DD at the 24-h time points might be reasonably linked by a dose bridging study. This is very compelling, because responses following NM recognition (Figure 9b) rely on changes in gene regulation and mRNA expression, which occur very fast and peak within the first hours [73,135]. This is particularly true for professional immune cells. Here, the decision-making about the subsequent induction of harmless immune tolerance or the initiation of vigorous defensive responses, eventually including excessive and harmful inflammation, irreversibly defines the final outcome. After this point, subsequent protein expression, cell-to-cell signalling via cytokine and chemokine secretion, cell differentiation, and the impairment of cellular viability due to toxicity are predetermined.

##### Metadata on Particle Agglomeration Relevant for In Vitro Bioassays

In the present case study, the set of information (data and metadata) listed in Table 4 emerged as relevant for characterisation of NP agglomeration. To ensure data FAIRness and reproducibility of the experimental readouts in bioassays, the relevant properties along with the accompanying metadata have been determined. These metadata are categorised into three data object groups: (i) data about the primary NPs, (ii) data about the agglomerates and (iii) data about the experimental environment such as cell medium conditions.

Concerning size information, for instance, using the mean diameter is just one option and has to be complemented by metadata providing information on size distribution and measurement method (e.g., hydrodynamic diameter versus dry diameter) [136]. Determination or reasoned assumptions on both mean and distribution is required to calculate the DD. Ensuring the availability of the necessary metadata alongside experimental data (offered by data creators) is essential to facilitate reuse by data customers. Without the metadata, interpretation, comparison, interoperability determination, and therefore the reusability of specific data about biological endpoints, such as exemplarily described in Figure 9b, is impossible.

NP agglomeration may have diametrically opposed consequences on different biological endpoints. For some biological endpoints, agglomeration has an inhibitory effect, for others it works as promoter, and this acts on top of particokineticly-driven DD variations. Inhibitory effects occur when agglomerate size increases beyond the size compatible for effective NP uptake by pinocytosis. In contrast, increased size is associated with improved particle uptake efficiency by phagocytosis although the shift of NP uptake from pinocytosis to phagocytosis may occur gradually [137] and is very different for cells of the phagocyte system or other cell types, like epithelial cells.

Additionally, NP agglomeration induces a change in the surface-to-volume ratio of the newly formed particles, hence, scaling down the particle quantity and modifying the quality of direct NP surface-to-cell membrane interactions. This is of special importance, because in general the NP surface area correlates more to cellular responses than the NP mass [138,139].

##### Essential Information Set for Bioassays Enabling Better Data FAIRness and Reproducibility

Data creators, experimentalists as well as modellers, involved in the early phases of the data life cycle (Figure 2), are the critical groups who have to provide information (data and metadata) on NP agglomeration. Due to the high sensitivity of NP agglomeration to small changes in experimental conditions, and protocols, as well as the specific properties of the used NPs, missing metadata cannot be easily curated at later stages increasing data uncertainty. Hence, missing metadata on the physicochemical characterisation undermines the reusability of the in vitro data by potential data customers and thus limits the value of the experimental outcome. However, not all information on NP agglomeration is of equal importance. Using a pragmatic approach for the requirements of bioassays, we can identify metadata from a continuum of physicochemical data on NP agglomeration:

- At one end of the continuum are (meta)data, which cannot be determined with reasonable effort within an experimental setup (e.g., shape, primary particle density). Experimental confirmation, however, could be replaced by well-reasoned suggestions for these without introducing a wide margin of error to the dataset and the experimental outcomes.
- At the other end of the continuum are (meta)data with a high impact on the confidence in the results from the respective bioassay, as they render the experimental outcomes sensitive to high variations (e.g., packing factor, effective density of agglomerates, agglomerate stability). Therefore, a thorough physicochemical characterisation is essential for these NP properties, and metadata have to include all information needed to facilitate for e.g., assessment of the in vitro DD or for in vivo-to-in vitro dose bridging studies.

The presented (meta)data continuum (Table 4) proposes a balanced set of information dataset that are essential for particle agglomeration to be defined.

All DD assessment models rely on detailed information on size, size distribution and effective density of primary particles in the relevant medium of the bioassay, as well as of agglomerates and possible changes over time. For primary particles, the effective density, especially for NMs with multiple components, advanced synthesis protocols, surface functionalisation and/or extended porosity or geometry is not readily available to the data creators and needs to be determined under the specific bioassay conditions. The situation is even more complex with agglomerated particles. The effective density of the agglomerate here depends on the packing model of primary particles and methods for determination may be more sophisticated. Information on agglomerate stability—this means ongoing changes in agglomerate size and size distribution during the time course of the in vitro experiment—is additionally needed and should be an essential part of a minimum (meta)data set.

#### 3.2.2. NMs Dissolution: Achieving Consensus on Terminology and Metadata Usage

We recognise that the sample size is small and from a narrow cross-section of colleagues who were probably offering a first impression of the survey tool’s questions. Hence, we present the results (see Table 3 in Section 2.2.2 for an overview of the suggested metadata) using a gradation of terms (consensus, near consensus, reasonable agreement, some agreement, and significant commentary) in summarising the results:

- Terms and definitions: There is consensus that the terms dissolution, solubility and leaching, are pertinent to nanosafety and warrant specific definitions for this field. Respondents either accepted the suggested definitions or offered improvements, but did not propose additional terms. There was less acceptance with the suggested visualisation of a NP, though it may yet be a useful tool in prompting a fuller description of the particle/nanoform under study. The visualisation was for a core-shell NP with a nanoscale surface coating. Further labelling and explanation are needed. Two colleagues viewed the dissolution definition as vague relative to that for melting.
- Suggested unit of measurement: There is near consensus that it would be desirable to have an accepted unit of measurement with caveats on normalising to particle surface area (flux) when measurements are usually reported as solution concentration. Those interested in therapy and toxicity focus more on the release of drug active or toxicant (dissolution rate in the questionnaire) and less so on poorly soluble carrier materials. Flux is more acceptable when the whole of the particle is of interest. The most relevant time scale will vary by experiment.
- Catalogue of competing reactions: There is consensus that there are competing reactions to be considered in the experimental design (no one challenged the concept and additional phenomena were suggested); reasonable agreement that the investigator should express their results in the form of a chemical reaction. The range of comments is similar to the distinctions made above for dissolution rate and dissolution flux.
- Induction time effects: There is consensus that the concept is pertinent to experimental design and nanoform architecture (NP structure). As with competing reactions, no one challenged the concept and several proposed additional sources of induction effects.
- Catalogue of media: Significant commentary from respondents that the media listed in the survey are prominent, but the number of suggestions for additional media implies that a general approach on listing metadata should be pursued over listing media. There was some agreement on listing media according to pathways, but the purpose for selecting a medium was paramount.
- Catalogue of current standardised methods: Significant commentary as a number of respondents were not aware of the extent of this listing, especially those with a nanosafety background not being aware of the test methods used in drug development.
- Catalogue of calculational models used for data interpretation: The listed models are pertinent, but significant commentary indicates that they are not used widely in the nanosafety community and still tied to the investigator’s disciplinary field rather than generalised.
- Five respondents mentioned silver as the material with the most complete data set and two proposed zinc oxide (Question 10c).

One ‘best in class’ journal article (Question 10b) was proposed by two respondents, and is pertinent to nanosafety as it centres on the pulmonary retention kinetics of barium sulphate NPs [140]. Examining the elements in the questionnaire with their counterparts in a peer reviewed article offers some insight into the challenges and benefits of formalising metadata capture within a FAIR context.

- Terms and Definitions: distinction between solubility and dissolution similar to the definitions used in the questionnaire. Many terms are used to express nuances: dissolution; ion-leaching; shedding of ions; released ions; dynamic (non-equilibrium); quasi-dynamic system; non-equilibrium; solubility; equilibrium; static (equilibrium); static-solubility; solubility limit; and supersaturation.
- Units of measurement: ng/cm^2^/h and %/day; stoichiometry used for chemical reaction.
- Competing reactions: not discussed as such. Terms used: binding events; secondary NPs; re-precipitation; recrystallisation; bioprocessing; transformation; modulation of biopersistence; and complex re-speciation.
- Induction time effects: not discussed as such, but indicated through terms that reflect the trajectory from under-saturation to supersaturation, such as: slowing onset of dissolution; saturation-related events; Ostwald ripening; and concentration limited. The length of time to be associated with a specific induction effect is not yet established.
- Media: not on the questionnaire’s list. Terms used are: physiological buffer; phagolysomal simulant fluid; receptor medium; dissolution buffers; eluates; simulant fluids; pH 4.5.
- Methods: flow-through method was on the questionnaire’s list; flow-by method is not. Terms include: flow-by abiotic and flow-by dialysis; and flow-through abiotic dissolution.
- Models: first order kinetics using stoichiometry to relate barium ion concentration in the existing medium to solid mass.

Keller et al. is an excellent illustration of the many concepts and nuances found in nanosafety. These colleagues’ focus is particle clearance from the lung, and they find it necessary to utilise many terms in distinguishing chemical dissolution from physical removal, e.g., mucociliary propulsion. Having a more formalised approach to metadata would lead to an economy regarding terms and would also focus the readers’ attention onto the specifics of modelling BaSO_4_ dissolution rather than on introducing the reader to the chemical kinetic nuances in their experimental design, especially in the case that Keller et al.’s results need to be re-interpreted as the field progresses. Similarly, it is also clear that a formalising metadata goals does avoid premature standardisation of test methods, i.e., metadata goals permit greater flexibility in medium selection.

In conclusion, the questionnaire has been useful for identifying the metadata required for dissolution studies in nanosafety and is a preliminary step in addressing FAIR and FAIRness scores for NM dissolution datasets. The metadata elements for a journal article or database are a controlled vocabulary (terms and definitions); a set of considerations to be addressed in the Materials and Methods section (media, method, competing reactions, and induction times); and interpretive tools (dissolution reaction, measurement unit, model).

The literature identified by respondents on separate instances can be found in references [140,141,142,143,144,145,146,147,148,149,150].

### 3.3. Conclusions on the Presented Case Studies

To test ways of forming consensus for metadata standards, the two presented case studies approached the issue from different perspectives:

1. Whether common metadata for both experimental and in silico workflows can be identified that can fully explain a phenomenon;
2. Whether consensus can be reached between experts from different scientific fields on a particular subject regarding the necessary metadata and approaches.

In the first instance, an in silico simulation of NP agglomeration identified a series of descriptors needed to explain the whole process. These descriptors were divided into two categories. Those (e.g., shape, primary particle density) that can be estimated with a reasonable error margin with no significant effect on the outcomes and those (e.g., packing factor, effective density of agglomerates, agglomerate stability) where lack of information will lead to significant errors, unreliable conclusions and low reusability. While for the former the lack of metadata can be overcome with computational approaches (e.g., data imputation) to increase reusability, the latter cannot be dealt with similarly. In their case, a clearly defined set of metadata needs to be identified and established as a pragmatic schema. This needs to include the necessary information to allow bridging between different types of study (e.g., in vitro vs. in vivo vs. in silico). One case study is definitely not able to completely achieve this, but it serves as a demonstrator to raise awareness of the importance of metadata and hopefully triggers more discussion.

Based on these observations, the second case study was designed to focus on the opinions of experts on the cross-disciplinary topic of NM dissolution. Dissolution is extensively studied by both experimentalists and computational scientists and is of key importance in all nano-related fields (e.g., (eco)-toxicology, nanomedicine, materials design). A characteristic example, as mentioned above, is the analogy between nanomedicine dissolution for the release of therapeutic agent and the toxicant shed by an NM in an e.g., environmental context. The survey results demonstrated consensus or near consensus on a wide range of issues including the terms and definitions to describe dissolution, units of measurements, the effects of the experimental design and the complexity of the process based on the provided catalogue of competing reactions. On the other hand, significant variance of opinion was observed on issues that can be considered more field specific like the experimental media (and the way these should be listed) and the standardised methods and analytical models.

These results demonstrate that cross-field data interoperability and reusability is possible when it comes to NM dissolution, with the caveat that further work is needed to bridge the differences between, for example, the nanosafety and nanomedicine communities. Consensus building needs to focus on the way the different experimental parameters, methods and analytical techniques are recorded and presented. This is especially true for the calculation models, as the responses demonstrated that these are still correlated with disciplinary fields. The more formalised and generalised metadata become, the less terms would be needed to explain different phenomena and the more general ontological terms to annotate the datasets to increase their FAIRness could be established, significantly decreasing the workload to achieve the necessary proof of data equivalence and interoperability.

## 4. Discussion on Metadata Challenges and Recommendations for the Nano-Community

### 4.1. Metadata Related Challenges for the Nano-Community Need Ongoing Attention and Mitigation

The fluidity and versatility of experimental and computational NMs research makes it hard to frame generalised recommendations applicable to the entire nanotechnology community. Metadata and its handling are an ongoing discussion, which has been addressed partly in previous NDCI papers [28,29] and is gaining increasing attention due to the pursuit of the FAIR data principles and the emergence and rapid evolution of nanoinformatics, which as discussed in the EU-US Roadmap Nanoinformatics 2030, provide a means for sorting, evaluating, comparing and analysing data for later reuse [40]. The continuously evolving computational methods require an increasing amount of high-quality FAIR data to uncover hidden patterns, which results in greater need for data interoperability that are currently not available. Lack of metadata is also a major reason for the reproducibility crisis [151,152], often caused by insufficient description of experimental details (so called pre-producibility [153]).

Here, we want to specifically highlight that even if it looks like a technical problem that can be addressed by the FAIR principles with further work [154], we are convinced that it cannot be solved by technology alone. Therefore, significant sections of the paper have focussed on the human factor, i.e., the need to build understanding and commitment of all players involved in the data lifecycle to improve the status quo, and the need to reach community-wide consensus on levels of metadata completeness making the data fit for reuse in broader settings. To achieve this, we must look beyond the original “technical” FAIR principles that are directed towards database owners and introduce key operational steps which data creators can take responsibility for. Reusability principle R1 “meta(data) have a plurality of accurate and relevant attributes” [33] is intentionally defined vaguely, creating a field-specific gap that each community needs to fill by defining its own guidelines in support of the original FAIR principles, i.e., “scientific” FAIR Principles. The moving target of metadata gold standards, also implicitly encoded in the “scientific” FAIR principles, described in Section 1.2 above, contradicts the idea of standards, which can be implemented into file templates or data upload interfaces once and then the user just has to follow these. Instead, constant evaluation is needed to ensure such templates continue to meet the requirements imposed by the community and the progress in our scientific discipline.

Based on the state-of-the-art presented above, even without universal standards, certain requirements and goals can be established with respect to metadata acquisition and availability. These can be used to promote further discussion to reach field-specific consensus on the needed metadata and terminology promoting translational research and enhancement regarding risk governance and the regulation of NMs. Taking account of the benefits of data sharing and, in that way, of metadata collection and management, a more organised approach with wider visibility is needed. From the current efforts mentioned above regarding relevant metadata schemas, only the MIBBI [95] and MIACA [155] are referenced in FAIRsharing.org. Taking into account the rapid technological and experimental evolution, MIACA can be considered outdated as it was last revised in 2008 and the available information is currently poorly suited to reuse from a biological standpoint [156]. MIBBI, on the other hand, was last updated in 2019, while the recent efforts of MIRIBEL [96] and MINBE [97] are not listed as FAIRsharing.org does not currently contain any nano-specific standards. This inhibits wider adoption from the community, implementation to everyday scientific research and thus data availability, interoperability and reusability, but may drive the adaption of community developed approaches.

### 4.2. (Meta) Data Generation and Capture Need to Be Implemented into DMPs along with the Use of Modern (Meta) Data Capturing Tools (ELNs)

Increasing computational power, coupled with the availability of clean, precise, annotated data and accompanying metadata, has led to the successful use of machine learning approaches for metadata analysis, identification of gaps, anomalies, data refinement and the uncovering of hidden patterns, as demonstrated by Labouta et al. (2019) [130]. The increased availability of such tools coupled with easier access and user-friendly interfaces in combination with the availability of more reference data, will dissolve the perceived separation between data creators and re-users. The incorporation of all the data and metadata needed to facilitate data re-use at the study design stage will help deciding on the most relevant experiments and identifying potential experimental and computational gaps. It will also speed up method development and customisation by integrating expertise from different fields and enriching empirical datasets with data or predictions from different sources. To be successful, these attempts need to be complemented with the use of tools (ELNs) that capture the related (meta)data during data generation, whether experimental or computational, with minimal, if any, losses resulting in datasets of higher quality. Furthermore, ELNs can assist with the implementation of FAIRness in the science itself. The development and integration in ELN of pre-annotated customised templates assisting with data generation and capture and guiding the user on (meta)data capture, will significantly decrease the workload for all the data roles presented in Table 1 and Table 2, and increase data availability, interoperability, and reusability, especially in a modern computational (i.e., machine and deep learning) context.

### 4.3. Relevant Databases Need to Make Metadata Submission Mandatory and Implement QA Processes

Current practices demonstrate that while there is willingness for a wider range of metadata provision and exploitation, these are still limited in many cases. Most current databases do not currently require detailed metadata in terms of experimental protocols, instruments and software used or computational workflows. In fact, one of the most difficult tasks of database managers is the gathering of detailed metadata. QA and QC practices are generally lacking or are left to the data creators and, partly, to curators or managers that may not have the necessary knowledge to evaluate the data being annotated. The lack of standardised protocols and reporting formats is a further burden. There are persistent claims that protocol standardisation, although helpful in terms of regulatory and governance practices, delay scientific progress and lead to constraints on innovation, but these are unfounded. True data sharing and re-use requires clear metadata standards, and the scientific FAIR principles presented here represent a first step.

### 4.4. Community Alignment Is Needed with Respect to Ontological Development

The use of standardised and linked ontologies, on the other hand, might overcome the hurdles to data reuse, but everyday practices also demonstrate a current lack of alignment leading to many parallel attempts rather than a clear consensus to expand and restructure (where necessary) existing ontologies. This is problematic from a computational point of view, where a more standardised approach in terms of terminology definition, equivalency and hierarchy is needed to further enhance interoperability and become less field-specific, a point firmly stressed in the EU-US Roadmap Nanoinformatics 2030. The formatting and annotation requirements of the computational community are distinctly different from those of the regulatory community. While both are cross-disciplinary, the data and metadata requirements are totally different. The robustness of calculated data for regulatory purposes are not fit for purpose for the structured, but fluid, nature of the modelling community [40]. The current publicly available data may be interoperable under a regulatory or scientific context, but require substantial processing to become computationally usable. This means several assumptions on metadata similarity or expansion of the dataset with relevant metadata is needed to identify statistical similarity or significant difference [124,125,126,127,128,129,130]. As a result, the currently existing databases do not meet the needs of the modelling community, which has led to the emergence of computational databases, allowing capture of computational descriptors and direct application of modelling tools [157]. For example, the NanoSolveIT project is developing a modelling-dedicated database through the Enalos cloud platform.

### 4.5. Community Consensus Is Needed to Promote Scientific and Technical Data FAIRness

Community consensus is needed to promote (meta)data FAIRness. This includes alignment in metadata reporting between different nano-related fields to harmonise the way (meta)data are captured, and reported. A good example is NMs dissolution, as highlighted in the case study presented here, which is of high interest for both the nanosafety and nanomedicine communities [40]. Dissolution is one of the more studied NM phenomena, which has led to a large volume of data being produced. These data, though, are not readily available for further exploitation and reuse, either due to being unavailable or being indexed under metadata or ontology rules other than those used in nanosafety research [29]. Building consensus, as demonstrated through this paper’s dissolution questionnaire, can lead to the unlocking, combination and reuse of a large volume of historical and current data for computational research. One thing to consider, however, is that the metadata needed to allow reuse across disciplinary boundaries is more comprehensive than the “minimum information” approach employed lately, as these are confined to supporting data reuse to address the specific research question for which the data was initially generated [29] and focus on identifying the test NM, rather than wider multidisciplinary use. The metadata requirements of the NIKC database, for example, far exceed the minimum required information and are closer to a complete reporting of all possible types of metadata.

The question is how consensus can be reached and who can lead these efforts. Ideally, this needs to happen at community level, i.e., through the EU NanoSafety Cluster, the EU NCI Working Group on Nanoinformatics (as per the current paper) or the EU-U.S. Communities of Research. In practice, the development of relevant case studies, i.e., dissolution and NM agglomeration, can spark the discussion and support the development of consensus. Indeed, this group is planning a follow-up consensus building activity and a subsequent publication.

### 4.6. Change in Scientific Mindset and Re-Education Is Needed to Support Implementation of the Scientific FAIR Principles

Addressing metadata collection systematically requires implementation of detailed management plans from the early design stages of experimental and computational practices. This also requires a change of mindset and a degree of re-education for all involved in modern experimental, computational and technical practices. Without a clear understanding of the needs and roles of the different parties and the benefits of harmonised collaboration, the much needed experimental, technical or data driven innovation will be unsuccessful. Data creators, analysts, curators, and managers cannot operate in isolation if they wish to meet the needs of data customers (data users and re-users). Thus, here, we proposed scientific FAIR principles, complementing the original technical FAIR principles, and a new data management role, that of the data shepherd to facilitate the communication between the different parties. The establishment, implementation and promotion of the scientific FAIR principles can only be successful if their benefits are clear to the entire community. To achieve this, training initiatives such as development of informatory massive open online courses (MOOC) courses, short courses and materials is needed by established stakeholders like NanoCommons, EMMC, EOSC. In any case, a transition period of redefining and restructuring data management practices is also needed, including further development of detailed publicly available DMPs (e.g NanoCommons DMP of which versions 1 and 2 are publicly available [132], with version 3 to be published shortly, including detailed dataset-specific exemplars for experimental, computational, and literature curated datasets).

### 4.7. Community Collaboration and Introduction of Data Shepherds Are Needed to Accelerate Progress

Achieving acceptable completeness and proven nanosafety data reusability, without increasing the workload of data creators to an unacceptable level, requires clear metadata capture workflows. Data reuse processes should ensure that this is done ethically and purposefully giving credit to the original data creators. Close cooperation between data creators, curators, managers and re-users, appropriate training at all these levels and the implementation of data shepherds, places us on the right track of stepwise progress towards the goal of having the required metadata for a multitude of reuse applications of the data in a standardised way. At the same time, we will be respecting the freedom of data creators to fully represent their data. While these roles are currently distinct, as discussed earlier, a significant part of data curation needs to move forward to the data creators that have all the required information, with dedicated curators having mainly the role of QC and guidance. Introduction of data shepherds who act as “translators” between the experimentalists, analysts and managers and are able to understand the requirements on all sides, will facilitate this transition to data management at the point of data capture. The interdisciplinary nature of the data shepherds and their ability to communicate with all data roles will help with training and promotion of understanding between all data roles. Of course, some time will be needed to operationally define the data shepherd, train the respective individuals and insert them at the centre of the data lifecycle as demonstrated in Figure 2. With time, the data shepherd will become a key part of the data ecosystem. Until then, their role needs to be covered, if possible, by dedicated data curators with the relevant training where possible. To achieve this, substantial effort is needed and commitment on all sides at various levels (individual researchers, project consortia, nanosafety community, policy bodies, funders) that can lead to the establishment of clear procedures and workflows, the necessary education and change of mentality and allow the data to reach its full potential of exploitation. Version 3 of the NanoCommons DMP will provide guidance on this.

### 4.8. User-Friendly Tools Need to Be Developed and Implement

The changes in daily practices of data generators noted in Section 4.7 should be technically supported by specific tools limiting manual operation and clearly structuring data collection and curation in order to reduce mistakes. One easily implementable step is the automation of the metadata collection process with the use of ELNs. Initially, such an approach requires manual handling and acquisition of the relevant details of instruments, protocols, NMs etc. In due time, it would be possible to automate the process by linking the ELN with instruments and software for the automated acquisition of the relevant settings used during data acquisition, with similar approaches possible to be implemented for simulation data, removing effort from researchers. Furthermore, the development of tools that allow experimentalists to use computational workflows (Jaqpot, KNIME, Jupyter notebooks etc.), will help experimentalists to appreciate the benefits of data science and assist in the promotion of (meta)data FAIRness and data sharing. These workflows can help turning metadata into data themselves to be used in metrology projects and read-across approaches (currently lacking for NMs), while at the same time speeding up research by removing manual entry of those pieces of information.

### 4.9. Recognise That Creating and Adhering to Community Standards Is Not Easy

Accomplishing the goals we describe in this paper will not be easy. A quote that puts the challenges of crating and using community standards into perspective is the following:

“Standards are like toothbrushes—everyone has one, but no one wants to use someone else’s.”

Phil Bourne, Former Associate Director for Data Science (Big Data), NIH

We think this is a very important reason for why we as individuals and as a community (or as sometimes disparate communities) struggle so much with metadata. Most of us likely find it much easier to start completely from scratch to build our own individual standard than to use an existing one. Our excuse is that the latter is not exactly what we need and it is often very hard or even impossible to get needed adaptations back into the standard and even if it is possible, it is then already too late since the project we were needing it for already moved on. We have attempted to include the flavor of this idea in this metadata paper, but perhaps it cannot be stressed enough. What we need to create and adhere to as a community is not a “metadata standard” but guidelines on what is needed and the call for action of the community to move the discussion from the technical aspects (templates in Excel, ISATAB, web interfaces, ...) to a way, how to get all of us in the community to report the needed metadata including everything we and the community think is essential. It is clear that we cannot support every single person individually but if projects decide on their own standards and make these publicly available, then there are ways to get this metadata into databases.

In accomplishing all this, an additional requirement should be that we avoid free text fields if they are not absolutely necessary. Having additional fields for comments for human users is always an option, but understanding the data, at least to an extent to judge if the data are useful or not, should not depend on these fields.

## 5. Conclusions

Data interoperability and reusability, under the FAIR data principles, is of the utmost importance for moving the nanotechnology, nanomedicine, and nanosafety fields forward and delivering on the promise expected from scientists, i.e., innovation that meets socioeconomic goals. Metadata will play an integral part in this and certain practices need to be established, acknowledging the domain-specific requirements, to facilitate metadata capture and reporting. This requires achieving a pragmatic gold standard, which is not the unrealistic capturing of complete metadata, but a fine balance among the workloads of all data-related roles, and especially data creators, the information captured and sufficient metadata to broaden the fields where reuse of the data will be feasible. These community metadata standards, aligned with scientific FAIR principles, need to be continuously refined based on the specific field and the technical and computational developments.

Clear DMPs covering the entire data lifecycle, and different types of data and metadata, should be defined and implemented from the very early stages of experimental or computational design. The DMP should incorporate clear templates for data usage requests in the form of metadata files or data upload tools implementing data validation steps. This can be achieved with specific and pragmatic workflows and the use of modern tools like ELNs, which will move data and metadata curation to data creators and allow them to incorporate it into their experimental design. While the set-up cost of ELNs can be high initially, it will shift and incorporate parts of the data processing, which data creators do anyway, from the end of scientific practice (e.g., data gathering, formatting, cleaning) to everyday experimental practice. This in turn will ensure that data curators have more time for quality control, increasing the overall utility and reusability of nanosafety data and the confidence of data customers. By working closely with data users and integrating their feedback on metadata completeness, and correcting any inconsistencies that become visible during reuse, the value of data will further increase. All these steps require a change of mentality and re-education of all involved (data creators, analysts, curators, managers, users and re-users) to facilitate these new processes. To support this transition, we propose a new role of data shepherds, who have knowledge and understanding of all aspects of the nanosafety data lifecycle, to help with the communication and sustainability of the model.

In the longer term, the systematic integration of nanosafety data will result in a focus on themes and topics that need further data coverage (data-driven funding) and can help with promotion of data-driven innovation, offering the potential for cross-community collaborations, more complex projects, advancement of translational research, and the establishment of a digital single market benefiting all. A call to action is needed within the nanosafety scientific community for the establishment of best practice and updating of lapsed or generation of missing standards to facilitate the process. This can be achieved through initiatives and close collaboration of the EU NanoSafety Cluster’s Working Group F on Data Management, the NCI Working Group on Nanoinformatics and other bodies like the Asian Nano-Forum. We hope that this paper will be the starting point for invigorating further discussion on the implementation of metadata and scientific FAIR principles for nanosafety data and the generation of further collaborative and consensus articles on, e.g., NM dissolution or the implementation and use of LIMS and ELNs in everyday scientific practice focussing on nanosafety and nanomedicine.

## Figures and Tables

**Table 1:** Data roles, responsibilities and interactions. Adapted from Hoover et al. [30,41] and Woodall et al. [42]. Adapted with permission from Elsevier, License Number: 4926970512437.

	Set objectives	Design Approach	Collect	Processing	Modelling/Analysis	Validate	Store	Share	Quality Control	Annotation	Determine Relevance	Apply	Confirm Effectiveness	Generalise	Communication and Education
Creators	X	X	X	X		X			X	X	X		X		X
Analysts		X		X	X	X			X	X	X		X	X	X
Curators				X		X			X	X	X		X		X
Managers							X	X	X				X		X
Customers	X								X		X	X	X	X	X
Shepherds	X	X	X	X	X	X	X	X	X	X	X	X	X	X	X

**Table 2:** Definition of the term “instance” for data creators, analysts, curators, managers, and customers.

Role	Example Term	Definition
Data creator	Experimental instance	A specific part of an assay or method
Data analyst	Training and test instances	A set of specific data entries used for training, testing and validating a predictive model
Data curator	NIKC instance	The reported nanomaterial in a system at a specific moment in time
Data manager	Database instance	A set of the background processes and memory structure needed by the database software to access the data
Data customer	All of the above depending on the specific use case	

**Table 3:** Suggested Metadata in the Dissolution Questionnaire.

Journal Article	Suggested Data/Metadata/Descriptors	Remarks
Introduction	Definitions for dissolution, dissolution rate, dissolution profile, and leaching; dissolution stoichiometry; potential for induction effects and competing reactions	Recommended to establish the study’s purpose relative to the literature
Materials & Methods	Apparatus relative to standardised test methods; Medium composition; stock dispersion shelf life and solution composition	Explanation that the chosen experimental design achieves the study’s purpose
Results—Reporting Units	mg/L/day for the analyte and ng/cm^2^/hr normalised to the particle surface area; initial and final surface images; and initial dissolution rates & solution compositions; final particle composition	As needed to address the experimental design and to allow for later interoperability and reuse.
Discussion	Computational model and characteristic dissolution rate and half-life.	Data analysis and interpretation should be related to study’s purpose
References	Sources of terms, apparatus, models	Sufficient to establish a basis for FAIR

**Table 4:** NP agglomeration-related metadata relevant for DD assessment as a means to facilitate correct interpretation of in vitro bioassays and support data reuse.

Data Object	NP Descriptors	(Meta)data	Remarks/Description	Case Study Value
Primary particle	Size	Diameter	Diameter of primary particle	50.0 ± 0 nm
		Determination method	DLS, NTA, TEM, SEM, …	NTA
		Statistical measure	Mean, mode, median, ...	Mean ± Stdev
		Size qualifier	Hydrodynamic diameter, dried, …	Hydrodynamic diameter
	Shape		Shape of particle (spherical, rod, …)	Spherical
	Aspect ratio		Ratio of sizes in different dimensions	1
	Density		Density of primary particle	SiO_2_: 2.2 g/cm^3^, TiO_2_: 4.24 g/cm^3^
	Surface charge		Zeta potential of primary particle	−34 mV *
	Porosity		Pore volume fraction	Non porous
	Polydispersity		Polydispersity index, size distribution	Monodisperse
	Dissolution rate		Release rate of molecular monomers and polymers	No significant dissolution
	Synthesis protocol		Protocol of particle synthesis/particle source	Reverse emulsion method: doi:10.1021/la052797 *
Agglomerate	Size	Diameter	diameter of agglomerate	250.0 ± 0 nm
		Determination method	DLS, NTA, TEM, SEM, …	NTA
		Statistical measure	Mean, mode, median, ...	Mean ± Stdev
		Size qualifier	Hydrodynamic diameter, dried, …	Hydrodynamic diameter
	Shape		Shape of agglomerate (spherical, rod, …)	Spherical
	Aspect ratio		Ratio of sizes in different dimensions	1
	Packing	Packing factor	Particle fraction of agglomerate volume (intra-agglomerate volume subtracted)	0.637
		Determination method	Volumetric Centrifugation Method, …	Default value: DOI:10.1186/1743-8977-7-36
	Effective density		Agglomerate density considering intra-agglomerate fluid	SiO_2_: 1.76 g/cm^3^, TiO_2_: 3.06 g/cm^3^
	Polydispersity		Polydispersity index, size distribution	Monodisperse
	Stability		Dissolution, agglomeration or dis-agglomeration over time	Stable
Experiment	Method		In vivo/in vitro/in silico	In silico
	Model		For in silico only: which method is simulated	In vitro, submerged, adherent cells
	Tool	Name	Tool name	ISDD
		Version	Software version	Current version
		Reference	Tool/method literature	DOI:10.1186/1743-8977-7-36
	Medium	Temperature		310 °K
		Viscosity		0.00074 Ns/m^2^
		Density		1 g/mL
		Type	Medium type (dH_2_O, PBS, serum free medium, …)	dH_2_O *
		Dish depth	Medium height level	0.25 cm
	Administered dose		Primary particle concentration administered	10 mg/mL
	Exposure time		Duration of NP exposure/simulation	24 h *
	Delivered dose		Primary particle mass deposited after exposure time	SiO_2_: 2.4 mg/cm^2^ * TiO_2_: 2.5 mg/cm^2^ *
	Dispersion protocol		Sample preparation details (sonication, …)	doi:10.3109/17435390.2012.666576 *

* Not relevant for in silico method.

## Supplementary Materials

The following are available online at https://www.mdpi.com/2079-4991/10/10/2033/s1, Metadata Questionnaire to Database Owners and Dissolution Questionnaire.
