# Supplementary material for: Metadata Stewardship in Nanosafety Research: Community-Driven Organisation of Metadata Schemas to Support FAIR Nanoscience Data
Source: Nanomaterials (Basel). 2020 Oct 15;10(10):2033. doi: 10.3390/nano10102033 (PMC7602672; doi:10.3390/nano10102033)
Supplement: Supplementary file 1 [file nanomaterials-10-02033-s001.pdf]

Supplementary Information for:

Metadata stewardship in nanosafety research: community-driven organisation of metadata schemas to support FAIR nanoscience data

Anastasios G. Papadiamantis[1,2,*], Frederick C. Klaessig[3], Thomas E. Exner[4], Sabine Hofer[5], Norbert Hofstaetter[5], Martin Himly[5], Marc A. Williams[6], Philip Doganis[7], Mark D. Hoover[8], Antreas Afantitis[2], Georgia Melagraki[2], Tracy S. Nolan[9], John Rumble[10,11], Dieter Maier[12], Iseult Lynch[1,*]

This manuscript, and all sections contained herein, were prepared for educational use. The views and assertions expressed in this volume are the private views of the authors and do not necessarily reflect, nor should they be construed as reflecting the views, and official policy of the Department of Defense, the Department of the Army, the U.S. Army Medical Department or the U.S. Federal Government. Further, use of trademarked name(s) does not imply endorsement by the U.S. Army but is intended only to assist in identification of a specific product.

Appendix 1. Individual responses to the metadata questionnaire provided by database owners

Note that Appendix has not been edited for language and format as we wanted to retain the inputs exactly as received from the questionnaire respondents.

Appendix 2: Dissolution Test Parameter (Metadata) Style Sheet

Appendix 1: Indiviidual as-received responses to the meetadata questionaire from database owners

Entry (ID 209)

Show empty fields

**Database name**	ACEnano Knowledge Infrastructure
**Link to website**	https://acenano.douglasconnect.com/
**First name**	
**Last name**	
**Email**	
**Position**	
**Is your database open or closed to submissions?**	Open
**Are data in the database public and reusable?**	Partially
**How is data reuse handled by the database? (e.g. referencing, credit)**	Accordingly to the Creative Commons Licence specified for each data workflow
**Do you DOI your datasets or use a unique referencing/ID system?**	Unique referencing / ID System
**Do you have a licensing system for your data?**	Yes
**You answered Yes to the**	Creative Commons https://creativecommons.org

**question above. Can you please tell us the licensing system(s) you are using?**	/licenses/
**Do the available data originate from extracted literature data, experimental data (e.g. raw, processed, from images, directly from instruments), computational or simulation data or from all three? Please tick all that apply.**	Experimental data
**How was/is the database populated?**	Both
**a. What are the re-use conditions?**	To respect the License mentioned for each Open Access data set. Additional information are included in the 'Terms of use' of the knowledge infrastructure https://acenano.douglasconnect.com/terms-of-use/
**b. Who owns the data once in the database?**	The Organisation that uploaded the data
**Does the database have a Quality Management System (e.g. ISO9000/9001)?**	No
**Is there a Data Management Plan (DMP) and if yes can you provide some details / a link?**	Yes, there is a Data Management Plan (DMP), but at the moment is a confidential report. An updated version will be available in the next 10-12 months.
**Do you use specific ontologies to annotate your data and metadata?**	Yes
**You answered Yes to the question above. Can you please tell us which**	EMBL-EBI Ontology Lookup Service https://www.ebi.ac.uk/ols/index that includes different ontology repositories

**ontologies you are using?**	
**Have you experienced difficulties due to different definitions of key terms?**	Yes
**You answered Yes to the question above. Can you please provide some relevant examples?**	No definition of terms / not available in any ontology repository, for example: rpm (revolutions per minute)
**Please rank your databases stance on meta-data description:**	Essential to have
**Taking into account the different types of metadata: bibliographical (dataset owner(s), contact information, etc.), descriptive (dataset abstract, ontologies used, revisions, data format etc.), technical (the methods and protocols used to produce the data, instrument details and settings), which types of metadata are included in your database? (Select all that apply)**	Bibliographical, Descriptive, Technical
**For the types of metadata included, do you have a metadata QA/QC tool (e.g. common system, unified methodology) to replace manual evaluation?**	Yes
**You answered Yes to the question above. Can you please provide some more details?**	For some fields when the metadata is collected we are using EMBL-EBI's Ontology Lookup Service as mentioned previously. However the difficulty of the user is to decide which terms to use when is listed in more than one repository.
**Does your database link to**	Yes

**underlying protocols used to generate the data?**	
**What are the main challenges you experience in relation to metadata?**	- Creation of inter-dependencies between different metadata information, when it becomes more complex<br>- Users do not always complete all metadata fields (reasons - time constrains, information not available, etc.). For this reason, not all metadata fields were made obligatory, so its completeness relies on owners's willingness to complete it.
**Do you have examples of best practice from your database or elsewhere that should be widely adopted? Please describe and add link / screenshot etc.**	This can be a good example: https://acenano.douglasconnect.com /data/analysis/53/ (the workflow describes the UV-VIS sample preparation, measurements, and data analysis of gold nanomaterial suspensions).<br>The process is explained in detail in the ACEnano Knowledge Infrastructure Manual: https://github.com /NanoCommons/tutorials/blob/master /ACEnano%20manuals/1.%20Cover%20page.md
**What do you do with metadata (handle, analyse, exploit)?**	Use for data selection / filtering, automatic analysis, included in the analysis reports
**What could you do with metadata that you are not currently doing? Are there any plans to work with the existing metadata (e.g. statistical analysis)?**	In progress: using the metadata for search in the entire database, additional selection and filters option, fully exploitation during the analysis especially when different data sets need to be combined and compared
**Can your database handle raw data / images / code etc.?**	Yes
**Do you consider that images could themselves be a type of metadata?**	No
**Would you consider integrating FAIRness scores for data into your database?**	Yes
**Are there any success stories regarding the use of metadata you would like to**	An initial example of a general workflow on how the metadata and data could be used: https://www.linkedin.com/pulse/workflow-collect-

share? analyse-report-nanomaterials-data-from-farcal/

Comments/Notes

Entry Details

Submitted: **Feb 28, 2020 @ 16:12**

Entry ID: **209**

Nanotechnology Working Group

Meta data questionnaire to database owners

A manuscript is under development by members of the Nanomaterials Data Curation Initiative (NDCI) that will explain to the greater informatics community the role of a metadata in the current efforts of data curation in nanotechnology. The NDCI was started within the National Cancer Institute's (NCI's) National Cancer Informatics Program's (NCIP's) Nanotechnology Working Group (Nano WG), but is open to the broader scientific community's participation.

Stakeholders for this paper include groups that currently curate nanomaterials data and metadata and strategic thinkers involved in developing best practices for metadata and data curation.

Please fill out the survey below to the best of your knowledge. Your response will contribute greatly toward a better understanding of the current state of utilisation of metadata as part of data curation for nanotechnology and will help advance the field of nanoinformatics.

Thank you!

Dear Iseult

We have dealt with a series of databases across several projects. As you are probably aware we have more recently, in pretty general terms, moved from using our own management database and data gathering templates (as discussed in our Nanosolutions/nanomile "collaboration", and via NSC presentations etc, etc) to use of the (more or less mandated) eNanoMapper database framework, though still using our templates, updated, and more aligned to the jrc ones, at the same time for experimental data gathering.

We have not answered the questionnaire for each different db, but have tried to give some more generic answers and observations on experiences etc, across these, that are currently relevant.

Filled in the Word questionnaire but the formatting went awry and tick boxes didn't work so some untidiness below. Hope it helps anyway.

1. Database name:

Several different ones from different projects. In three main groups:

1. Older ones that we in the past used our own database for administering and "assembling" templated Nano-EHS data, that have since been, to a greater or lesser extent imported to the eNanoMapper framework/database. eg ENPRA and MARINA, and we have shared data with other projects such as NanoReg & CaliBrate;
2. More recent closed ones that we worked on, that still at least theoretically have data embargoes that will be imported once agreement is obtained from data owners : eg SUN, NanoSolutions
3. Ongoing ones that will be put in to eNanoMapper databases in the project, (eg PATROLS, GRACIOUS, NANOINFORMATIX) with the aim of sharing by agreement, when reached/available.

2. Link to website:

See the above various project's websites, and https://search.data.enanomapper.net/

3. Survey completed by (name, email, position):

4. Is your database open or closed to submissions?

Both, depending on the project. Currently vast majority of data is closed-available only to relevant project members.

☐ X Open – Yes

☐ X Closed -- Yes

5. Are data in the database public and reusable?

☐ Yes –

☐ No

☐ **X Partially – --- Yes some MARINA data in the public eNanoMapper; ENPRA too, likely soon**

6. How is data reuse handled by the DB (referencing, credit)?

**Has been for individual projects to date, or through particular arrangements being made .**<br>**In general don't think these aspects are (sufficiently, if at all) implemented in current databases, either procedurally or technically**

7. Do you DOI your datasets or use a unique referencing/ID system?

☐ Unique referencing/ID system

☐ DOI

☐**X Both**

☐ None of the above – **Not in the older closed database. But will as the older data is uploaded to eNM Yes in eNM generally. For other project data, not to date but will be doing in new data projects.**

8. Do you have a licensing system for your data? If yes, which one do you use?

**In general for the projects not currently.**<br>**For eNanoMapper for public data - Yes**

9. Do the available data originate from extracted literature data, experimental data (e.g. raw, processed, from images, directly from instruments), computational or simulation data or from all three?

Please tick all that apply.

☐ Extracted literature data

☐ **X Experimental data**

☐ Simulation or computational data

10. For extracted literature data, is the extraction a passive or interactive process?

☐ Passive

☐ Interactive

☐ Both

11. How was/is the database populated?

☐ 3rd parties

☐ **X Internally**

☐ Both

12. Do you use a standardised curation method? Please provide some details.

Is there a standardised curation method?
Not sophisticated to date: Have used IOM FP7 templates in recent projects, and now also some JRC NanoReg templates. Templates and meti information gathered together into "admin" database and selected data extracted to related "results" database. The latter now being done via the eNM database parsing and loading

13. Is the data in the database FAIR (Findable, Accessible, Inter-operable and re-usable)?

☐ **Some - Yes**

☐ **Lots of the older – Strictly, No**

a. What are the re-use conditions?

To date, not standardised, & pretty much by arrangement via managers and interested parties

b. Who owns the data once in the database?

Generally the consortia / partners – generally it is hoped that more will become "public; but who then is the guardian? Esp. if sustainability and maintenance are required

14. Does the database have a Quality Management System (e.g. ISO9000/9001)? If yes, which one?

No

15. Is there a Data Management Plan (DMP) and if yes can you provide some details / a link?

For current projects yes. Not yet publicly available currently

16. Do you use specific ontologies to annotate your data and metadata?

☐ **x Yes**

☐ **x No Hitherto**

a. If yes, which ones?

Now, using eNanoMapper, use that ontology

17. Have you experienced difficulties due to different definitions of key terms?

☐ **X Yes**

☐ **No**

a. If yes, please give examples

18. Please rank your databases stance on meta-data description:

☐ **X Essential to have**

☐ Nice to have

☐ Don't mind if have or not

19. Taking into account the different types of metadata: bibliographical (dataset owner(s), contact information, etc.), descriptive (dataset abstract, ontologies used, revisions, data format etc.), technical (the methods and protocols used to produce the data, instrument details and settings), which types of metadata are included in your database? (Select all that apply)

☐ **X Some Bibliographical**

☐ **X Descriptive**

☐ **X Technical**

☐ None of the above

20. For the types of metadata included, do you have a metadata QA/QC tool (e.g. common system, unified methodology) to replace manual evaluation?

☐ **X Yes to some extent as principles but also**

☐ **X No not as a formal tool – minimum requirements QA/QC are developed and applied but partial use due to workloads and time required I believe**

a. If yes, please describe / give details

21. Does your database link to underlying protocols used to generate the data?

☐ **X Yes**

☐ No

22. What are the main challenges you experience in relation to metadata?

Finding, mapping & implementing relevant ontologies

Revision control

Collecting data in standardized format v wide variety of data formats used/required for the diversity of the research

Missing required information e.g. SOPs, instrument details, calc methods etc,

Lack of standard methods for QA/QC and the high overhead of their application anyway (which is generally underappreciated by the lab scientists (cc the data stewards)

23. Do you have examples of best practice from your database or elsewhere that should be widely adopted? Please describe and add link / screenshot etc.

Data inventory survey at start of the project both for formal DMP initially and data management planning, workflows and administration in the lifecycle of the project more generally

Overhead time for good curation and QA/QC still underappreciated and under resourced. Versioning can be problematic. In ENM now can use free tools like Phabricator to help control it.

24. What do you do with metadata (handle, analyse, exploit)?

Recording, Storing, retrieving, analysing, modelling, reporting, Data management itself.

E.g. use in data collection templates for labelling/linking to ontologies etc. Better search indexing for findable data.

25. What could you do with metadata that you are not currently doing? Are there any plans to work with the existing metadata (e.g. statistical analysis)?

High quality "intelligent" metadata where it is available could greatly enhance reuse of data for analysis, modelling, grouping, read-across, safe-by-design frameworks etc.

26. Can your database handle raw data / images / code etc.?

☐ **Yes – Older databases are flexible and could be adapted to include or directly link to raw data & image files etc.**

☐ No

27. Do you consider that images could themselves be a type of metadata?

☐ **Yes – Yes (can contains information where, when it was captured and also meta information about the image itself in image-file-inherent metadata (if preserved by the technology/workflow); Some are results in themselves, and may be scaled, many/some more qualitative in nature**

☐ No

28. Would you consider integrating FAIRness scores for data into your database?

☐ **Yes – Probably for the majority; It is certainly anticipated that in time we will use such -anyway**

☐ No

☐ Don't know

29. Are there any success stories regarding the use of metadata you would like to share?

**Successfully migrated data collected in FP7 projects (MARINA, ENPRA) to the eNanoMapper database using eNM ontologies & metadata. Not perfection, but a good large undertaking - still much in progress.**

Entry (ID 4)

Show empty fields

**Database name**	Biomax
**Link to website**	https://ssl.biomax.de/nanocommons/
**First name**	
**Last name**	
**Email**	
**Position**	
**Is your database open or closed to submissions?**	Open
**Are data in the database public and reusable?**	Yes
**How is data reuse handled by the database? (e.g. referencing, credit)**	Each data entry has an internal unique identifier that can be used for referencing. Also, each data entry have associated information about external references and IDs. Each data entry can have multiple entries for external DOIs. Each data entry also has associated depositor and full audit information. Every change in the system is tracked, with the timestamp, user-information, and the actual change performed.
**Do you DOI your datasets or use a unique referencing/ID system?**	Unique referencing / ID System
**Do you have a licensing**	Yes

**system for your data?**	
**You answered Yes to the question above. Can you please tell us the licensing system(s) you are using?**	Biomax does not own the data deposited in the Knowledge library. The data are either open or owned by original depositor.
**Do the available data originate from extracted literature data, experimental data (e.g. raw, processed, from images, directly from instruments), computational or simulation data or from all three? Please tick all that apply.**	Extracted literature data, Experimental data
**For extracted literature data, is the extraction a passive or interactive process?**	Both
**How was/is the database populated?**	Both
**Do you use a standardised curation method? Please provide some details.**	Data curation is performed by the NanoCommons consortium members, according to the established NanoCommons data curation process.
**Is the data in the database FAIR (Findable, Accessible, Inter-operable and re-usable)?**	Yes
**a. What are the re-use conditions?**	As each data entry contains a unique identifier, it is possible to cite the data properly, and the conditions of use are clearly communicated. Reuse is managed by the NanoCommons Data Management Plan and the licenses of the data sets.
**b. Who owns the data once in the database?**	Biomax provides the technology, infrastructure and hosting of the data. The data themselves are either

	open or owned by the original depositor.
**Does the database have a Quality Management System (e.g. ISO9000/9001)?**	Yes
**You answered Yes to the question above. Can you please tell us which Quality Management System you are using?**	ISO 9001 and ISO 27001
**Is there a Data Management Plan (DMP) and if yes can you provide some details / a link?**	Yes, it is managed by the NanoCommons Data Management Plan.
**Do you use specific ontologies to annotate your data and metadata?**	Yes
**You answered Yes to the question above. Can you please tell us which ontologies you are using?**	These are the ontologies that are currently implemented: eNanoMapper Ontology, NanoParticle Ontology, MeSH Ontology, NCI Ontology, Gene Ontology, PATO Ontology. Furthermore, we can very quickly implement any other, existing ontology or even a NanoCommons-specific ontology.
**Have you experienced difficulties due to different definitions of key terms?**	No
**Please rank your databases stance on meta-data description:**	Essential to have
**Taking into account the different types of metadata: bibliographical (dataset owner(s), contact information, etc.), descriptive (dataset**	Bibliographical, Descriptive, Technical

**abstract, ontologies used, revisions, data format etc.), technical (the methods and protocols used to produce the data, instrument details and settings), which types of metadata are included in your database? (Select all that apply)**	
**For the types of metadata included, do you have a metadata QA/QC tool (e.g. common system, unified methodology) to replace manual evaluation?**	No
**Does your database link to underlying protocols used to generate the data?**	Yes
**What are the main challenges you experience in relation to metadata?**	As the data sets deposited in the Knowledge library come from different research groups, keeping it uniform across the domain while still allowing the depositors to have the freedom they need to fully represent their data is the biggest challenge.
**What do you do with metadata (handle, analyse, exploit)?**	All metadata in the Knowledge library is fully searchable, categorizable, analyzable and usable. The meta-data can be used, integrated, linked, analyzed and manipulated fully. There is virtually no limitations as to what can be done with the metadata.
**What could you do with metadata that you are not currently doing? Are there any plans to work with the existing metadata (e.g. statistical analysis)?**	This depends on the needs of the NanoCommons partners. We are currently implementing statistical analysis method. We could implement image analysis and expression analysis pipelines.
**Can your database handle raw data / images / code etc.?**	Yes
**Do you consider that images could themselves be a type of metadata?**	Yes

**Would you consider integrating FAIRness scores for data into your database?**	Yes
**Are there any success stories regarding the use of metadata you would like to share?**	NanoMile and NanoFase projects

Comments/Notes

Entry Details

Submitted: **Mar 13, 2019 @ 14:07**

Entry ID: **4**

Entry (ID 216) Show empty fields

**Database name**	NanoInformatics Knowledge Commons
**Link to website**	http://nikc.egr.duke.edu
**First name**	
**Last name**	
**Email**	
**Position**	
**Is your database open or closed to submissions?**	Open
**Are data in the database public and reusable?**	Partially
**Do you DOI your datasets or use a unique referencing/ID system?**	None of the above
**Do you have a licensing system for your data?**	No
**Do the available data originate from extracted**	Extracted literature data, Experimental data

**literature data, experimental data (e.g. raw, processed, from images, directly from instruments), computational or simulation data or from all three? Please tick all that apply.**	
**For extracted literature data, is the extraction a passive or interactive process?**	Both
**How was/is the database populated?**	Internally
**Do you use a standardised curation method? Please provide some details.**	Yes, We use the NanoInformatics Knowledge Commons - Instance Organizational Structure (NIKC-IOS), which informs how we structure the data that are curated into the NIKC database. Researchers populate an Excel Template incorporating the NIKC-IOS, which enables users to capture experimental and bibliographic metadata. The NIKC database was designed to capture as much metadata is necessary to make curated datasets re-usable. The data is mapped before it is uploaded into the NIKC Excel template to help organize data and metadata in to the NIKC-IOS.
**Is the data in the database FAIR (Findable, Accessible, Inter-operable and re-usable)?**	No
**a. What are the re-use conditions?**	We currently do not have re-use conditions.
**b. Who owns the data once in the database?**	The NIKC database is meant to be a repository for researchers' datasets. The individual or individual's organization continues to own the data once uploaded onto the database. Any use or sharing of the data must be permitted by the curator of the data, even with other NIKC database users.
**Does the database have a Quality Management System (e.g. ISO9000/9001)?**	No

**Do you use specific ontologies to annotate your data and metadata?**	Yes
**You answered Yes to the question above. Can you please tell us which ontologies you are using?**	eNanoMapper<br>snomed<br>National cancer institute thesaurus<br>uniprot (technically not an ontology; reliable reference to identify organisms[Latin name and strain], proteins, and genes)<br>cell line ontology<br>Ontology for Biomedical Investigations<br>chebi<br>International Organization for Standardization (not an ontology)<br>BRENDA Tissue Enzyme Source Ontology<br>Nanoparticle ontology<br>USDA soil classification (not an ontology)<br>medical subject headings<br>Cell Culture Ontology<br>and others
**Have you experienced difficulties due to different definitions of key terms?**	Yes
**You answered Yes to the question above. Can you please provide some relevant examples?**	A broad example would be terms that appear in multiple ontologies with varying definitions based on context. Sometimes the correct term can be found under a different context resulting in a different definition.
**Please rank your databases stance on meta-data description:**	Essential to have
**Taking into account the different types of metadata: bibliographical (dataset owner(s), contact information, etc.), descriptive (dataset abstract, ontologies used, revisions, data format etc.), technical (the methods and protocols used to produce the data, instrument details and settings), which types**	Bibliographical, Descriptive, Technical

**of metadata are included in your database? (Select all that apply)**	
**For the types of metadata included, do you have a metadata QA/QC tool (e.g. common system, unified methodology) to replace manual evaluation?**	No
**Does your database link to underlying protocols used to generate the data?**	No
**What are the main challenges you experience in relation to metadata?**	When curating from literature, publications do not have set standards regarding nanomaterial characterization data. The authors decide how to describe their nanomaterial leading to, some publications with very little to no characterizations, while other publications have detailed characterizations.<br><br>When working with researchers to curate experimental data, the amount of metadata captured is often limited to available funding. Researchers also tend to collect just enough metadata necessary to analyze their data according to their experimental questions. This results in similar datasets with varying degrees of metadata collected.
**Do you have examples of best practice from your database or elsewhere that should be widely adopted? Please describe and add link / screenshot etc.**	We are still developing guidelines for best practices.
**What do you do with metadata (handle, analyse, exploit)?**	We are currently using metadata for app development.
**What could you do with metadata that you are not currently doing? Are there any plans to work with the existing metadata (e.g. statistical analysis)?**	Our plan is eventually use metadata for analysis (statistical, machine learning, best practices for regulation, further app development, and assay development).

**Can your database handle raw data / images / code etc.?**	Yes
**Do you consider that images could themselves be a type of metadata?**	Yes
**Would you consider integrating FAIRness scores for data into your database?**	Yes

Comments/Notes

Entry Details

Submitted: **Apr 29, 2020 @ 14:58**

Entry ID: **216**

Entry (ID 9) Show empty fields

Database name Safe & Sustainable Nanotechnology (S2NANO) DB

Link to website http://portal.s2nano.org/

First name

Last name

Email

Position

Is your database open or closed to submissions? Closed

Are data in the database public and reusable? Partially

How is data reuse handled by the database? (e.g. referencing, credit)

We request users of our data to reference the DB portal (s2nano.org) and published articles [1-3] [1] Ha, M. K., Trinh, T. X., Choi, J. S., Maulina, D., Byun, H. G., & Yoon, T. H. (2018). Toxicity classification of oxide nanomaterials: effects of data gap filling and PChem score-based screening approaches. Scientific reports, 8(1), 3141.
[2] Trinh, T. X., Ha, M. K., Choi, J. S., Byun, H. G., & Yoon, T. H. (2018). Curation of datasets, assessment of their quality and completeness, and nanoSAR classification model development for metallic nanoparticles. Environmental Science: Nano, 5(8), 1902-1910.
[3] Jang-Sik Choi, My Kieu Ha, Tung Xuan Trinh, Tae Hyun Yoon, Hyung-Gi Byun (2018) Towards a generalized toxicity prediction model for oxide nanomaterials using integrated data from different

sources. Scientific reports, 8(1), 6110.

**Do you DOI your datasets or use a unique referencing/ID system?**	None of the above
**Do you have a licensing system for your data?**	No
**Do the available data originate from extracted literature data, experimental data (e.g. raw, processed, from images, directly from instruments), computational or simulation data or from all three? Please tick all that apply.**	All of the above
**For extracted literature data, is the extraction a passive or interactive process?**	Passive
**How was/is the database populated?**	Both
**Do you use a standardised curation method? Please provide some details.**	We have used specific physicochemical (PChem) score screening and nano-specific data gap filling method proposed by S2NANO for data curation [1,2,3]. The PChem score screening system evaluates the quality and completeness of PChem data while the nano-specific data gap filling method replaces missing values with manufacturer's specifications and/or estimations.The quality and completeness of PChem data were determined by a set of rules that specifically gave a score for each PChem attribute (i.e. core size, hydrodynamic size, surface charge and specific surface area). The PChem score for each attribute is composed of two sub-scores; one for the reliability of the data source and another for the reliability of the measurement method. [1] Ha, M. K., Trinh, T. X., Choi, J. S., Maulina, D., Byun, H. G., & Yoon, T. H. (2018). Toxicity classification of oxide nanomaterials: effects of data gap filling and PChem score-based screening

approaches. Scientific reports, 8(1), 3141.
[2] Trinh, T. X., Ha, M. K., Choi, J. S., Byun, H. G., & Yoon, T. H. (2018). Curation of datasets, assessment of their quality and completeness, and nanoSAR classification model development for metallic nanoparticles. Environmental Science: Nano, 5(8), 1902-1910.
[3] Jang-Sik Choi, My Kieu Ha, Tung Xuan Trinh, Tae Hyun Yoon, Hyung-Gi Byun (2018) Towards a generalized toxicity prediction model for oxide nanomaterials using integrated data from different sources. Scientific reports, 8(1), 6110.

**Is the data in the database FAIR (Findable, Accessible, Inter-operable and re-usable)?**	No
**Does the database have a Quality Management System (e.g. ISO9000/9001)?**	No
**Is there a Data Management Plan (DMP) and if yes can you provide some details / a link?**	N/A
**Do you use specific ontologies to annotate your data and metadata?**	No
**Have you experienced difficulties due to different definitions of key terms?**	Yes
**You answered Yes to the question above. Can you please provide some relevant examples?**	Size : some authors did not clearly indicate their definition of size and use this term ambiguously, such as core (primary) size vs. hydrodynamic size, agglomerated or aggregate size... etc.
**Please rank your databases stance on meta-data description:**	Essential to have

**Taking into account the different types of metadata: bibliographical (dataset owner(s), contact information, etc.), descriptive (dataset abstract, ontologies used, revisions, data format etc.), technical (the methods and protocols used to produce the data, instrument details and settings), which types of metadata are included in your database? (Select all that apply)**	Bibliographical, Descriptive, Technical
**For the types of metadata included, do you have a metadata QA/QC tool (e.g. common system, unified methodology) to replace manual evaluation?**	Yes
**You answered Yes to the question above. Can you please provide some more details?**	We have a scoring system for QC of metadata, such as INFO score from bibliographical metadata (journal name, journal information, etc.), PChem and Tox scores from technical metadata (the methods and protocols used to produce the data, instrument details and settings)
**Does your database link to underlying protocols used to generate the data?**	No
**What are the main challenges you experience in relation to metadata?**	In our experience, the completeness and quality of data are challenges. Concerns for data completeness and quality are not only for physicochemical data of nanomaterials but also for in vitro toxicity data. The completeness of data is referred to missing data that some groups perform experiment to measure a parameter, but other groups do not. Concerns of data quality is related to standard measurements (e.g. Good Laboratory Practice, ISO protocols) that not all groups would refer to.
**What do you do with metadata (handle, analyse, exploit)?**	We mostly use our metadata for pre-processing purpose, to generate datasets for predictive model development. For example, we use metadata to sort, filter and screen original dataset to generate higher quality datasets or fit-for-purpose datasets.

**What could you do with metadata that you are not currently doing? Are there any plans to work with the existing metadata (e.g. statistical analysis)?**	We hope to expand current collection of metadata in terms of scope as well as quantity, and would like to perform exploratory data analysis for new model developments.
**Can your database handle raw data / images / code etc.?**	Yes
**Do you consider that images could themselves be a type of metadata?**	No
**Would you consider integrating FAIRness scores for data into your database?**	Yes

Comments/Notes

Entry Details

Submitted: **Apr 6, 2019 @ 8:05**

Entry ID: **9**

Entry (ID 7) Show empty fields

**Database name**	RIVM - ECOTOX
**Link to website**	Not available yet
**First name**	
**Last name**	
**Email**	
**Position**	
**Is your database open or closed to submissions?**	Closed
**Are data in the database public and reusable?**	Yes
**How is data reuse handled by the database? (e.g. referencing, credit)**	The database contains all refererences to the Original research papers and all references are publically available. It is to be noted that the database will be made available within e-Nanomapper.
**Do you DOI your datasets or use a unique referencing/ID system?**	None of the above
**Do you have a licensing system for your data?**	No

**Do the available data originate from extracted literature data, experimental data (e.g. raw, processed, from images, directly from instruments), computational or simulation data or from all three? Please tick all that apply.**	Extracted literature data, Experimental data
**For extracted literature data, is the extraction a passive or interactive process?**	Interactive
**How was/is the database populated?**	Internally
**Do you use a standardised curation method? Please provide some details.**	Each database ntry is manually checked. However, no strict criteria have been applied.
**Is the data in the database FAIR (Findable, Accessible, Inter-operable and re-usable)?**	No
**a. What are the re-use conditions?**	None - once the database is exported to e-Nanomapper anyone can use the data.
**b. Who owns the data once in the database?**	The database i currently owned by RIVM but will be made publically available.
**Does the database have a Quality Management System (e.g. ISO9000/9001)?**	No
**Is there a Data Management Plan (DMP) and if yes can you provide some details / a link?**	No

**Do you use specific ontologies to annotate your data and metadata?**	No
**Have you experienced difficulties due to different definitions of key terms?**	No
**Please rank your databases stance on meta-data description:**	Nice to have
**Taking into account the different types of metadata: bibliographical (dataset owner(s), contact information, etc.), descriptive (dataset abstract, ontologies used, revisions, data format etc.), technical (the methods and protocols used to produce the data, instrument details and settings), which types of metadata are included in your database? (Select all that apply)**	Bibliographical, Technical
**For the types of metadata included, do you have a metadata QA/QC tool (e.g. common system, unified methodology) to replace manual evaluation?**	No
**Does your database link to underlying protocols used to generate the data?**	No
**What are the main challenges you experience in relation to metadata?**	The main challenge is simply: to obtain the metadata
**What do you do with metadata (handle, analyse,**	These are included in the database as separate entries.

exploit)?

**What could you do with metadata that you are not currently doing? Are there any plans to work with the existing metadata (e.g. statistical analysis)?**	Fill in data gaps.
**Can your database handle raw data / images / code etc.?**	No
**Do you consider that images could themselves be a type of metadata?**	No
**Would you consider integrating FAIRness scores for data into your database?**	Yes

Comments/Notes

Entry Details

Submitted: **Apr 3, 2019 @ 12:47**

Entry ID: **7**

Dissolution Test Parameter (Metadata) Style Sheet

Appendix 2: Integrated responses to the Dissolution Questionnaire

Introduction: **~75 approached; 18 responses**

Dissolution testing is of great importance in characterizing nanomaterials. Currently, however, many results in the literature have limited value as the testing parameters, which we call test metadata, are not reported fully, leading to limited reproducibility, applicability and interoperability.

This is 11-page questionnaire focuses on nanomaterial dissolution testing under abiotic conditions with the expectation that the results will be found informative in an environmental, health and safety (EHS) perspective. Additional testing more specific to a regulatory review or to a particular nanomaterial application may be yet required, but we believe that accurate and complete reporting under abiotic conditions provides a necessary foundation.

The questionnaire has five parts, each dealing with an important set of information useful in reporting dissolution testing results:

1. Introduction and Conclusion regarding general information;
2. General (stoichiometric relationships); Definitions; Units;
3. Competing phenomena; Sample preparation; Dissolution media; Induction time;
4. Apparatus/Technique metadata; and
5. Data analysis.

The questionnaire's results will be analyzed and published in a paper on metadata challenges in nanoinformatics. The questionnaire results will be made available to all respondents. Further use in preparing a community of research consensus on dissolution metadata is also possible.

For that analysis, it would be helpful to know who our respondents are and their backgrounds. Please choose an option:

A). Anonymous; **One**

B). Name and Background, but not for attribution; or **Nine**

C). Name and Background with attribution. **Eight**

Dissolution Test Parameter (Metadata) Style Sheet

(1) General compositional and reaction data:

Dissolution contributes to both the therapeutic and toxicological effects of nanomaterials. The physical model below is used to differentiate the particle core from any coatings with a different chemical composition or layers of adsorbed species from post-manufacturing steps or environmental & test media constituents.

Additionally, the dissolution products being measured analytically may not have the same chemical composition as the solid. The solid may be a multi-component mixture (drug formulation); the dissolution products may dissociate, hydrolyze, oxidize and oligomerize; and there may chemical reactions that form a new layer or coating or that form additional solids in solution.

Preferred metadata to describe composition and reaction(s): the stoichiometric relationship between the dissolution product being measured and the source chemical composition in the solid should be stated explicitly as a chemical formula.

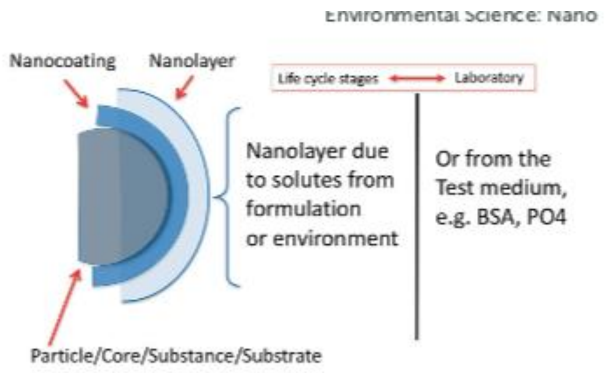

Questions:

(1a) Should investigators report a dissolution reaction equation that relates solution species to the original solid? **16 Yes; 2 No**

(1b) Is the physical model above an acceptable description of nanoparticles ? **10 Yes; 2 No**

(1c) Comments—suggestions – explanation – edits are welcomed.

(2). <u>Definitions of dissolution-related terms</u> – These are the suggested preferred terms and definitions

(2-1). Dissolution: the change in state (phase) of a chemical substance from a solid (solute) into a solution (solvent).
Note 1: Most pertinent when the chemical composition is the same in both phases.
[Combination of several ISO definitions]

(2-2) Dissolution rate: change of the dissolved mass of a solute with time.
[ISO 17327-1:2018 Non-active surgical implants - implant coating, Term 3.7]

(2-3). Dissolution profile: dissolution rate as a function of time

(2-4) Leaching: extraction of one or more constituents of a solid by a solvent
Note 1: Most pertinent with incongruent dissolution in a multicomponent solid
[modification of Term 2.5, ISO 16797:2004 Nuclear Energy]

(2-5). Solubility: maximum mass of a solute that can be dissolved in a unit volume of solution measured under equilibrium conditions.
Note 1: Specific to the solution composition
[ISO 17327-1:2018 Non-active surgical implants - implant coating, Term 3.16]

Questions:

(2a) Do the terms dissolution, dissolution rate, dissolution profile, and leaching each describe dissolution phenomena? **16 Yes; 2 No**

(2b) What is missing? **Dissolution rate constant; melting, dissolving, in vitro drug release; half time.**

(2b) Comments – edits – suggested terms – points of significant differences are welcome.

(3). Reporting Units

Dissolution rate and leaching rate:

- mg/L for the time period of a standardized test
- mg/L/day for the analyte being measured
- ng/cm2/hr as a normalized flux of the chemical composition in the solid

Preferred metadata: standard molarity or molality units for solution concentrations; and dissolution rate and dissolution flux for the dissolution reaction.

(3a). Do you agree that dissolution rate should refer to the analyte being measured and dissolution flux refer to the solid that is dissolving?

11 Yes; 7 No

(3b). Comments – suggestions – edits are welcomed.

(4). Competing mechanisms

- The measured solubility may deviate from thermodynamic limits for simple acidic and basic solutions due to:
  - Complexation due to chelants, ligands, and other constituents from the solution or due to oligomerization and other forms of speciation in dilute solutions;
  - Kinetic limitations due to adsorbed ligands and to any changes in chemical composition at the surface
  - Kinetic limitations due to dissolution mechanisms changing as the degree of under-saturation is varied
  - Standard thermodynamic calculations are for 'free' material, meaning uncomplexed species.

- In dynamic situations, where there is transport of the particle or of solution past the particle, there may be changes in surface chemistry due to variable adsorption of ligands, e.g. Vroman effect with proteins.

Preferred metadata: dissolution experiments should anticipate adsorption phenomena as part of the experimental design, consider adding potential adsorbates as experimental controls or refer to documentation where this was done.

Questions:

(4a). Is there a missing competing mechanism? **11 Yes; 7 No**

(4b) Comments are welcomed.

(5). Sample prep/stock dispersion

The focus here is on dissolution. It is expected that each physicochemical measurement, e.g. zeta potential, will have a specific set of metadata requirements, which we are terming a 'module.'

- Sample to be described in 'sample metadata module
- Stock dispersion preparation to be described in 'dispersion protocol module'
- Materials arriving in dispersion form to be described in the 'sample metadata module'
- Shelf life & the elapsed time since stock dispersion preparation should be metadata in the 'assay metadata module'
- The sample's dissolved concentration in the stock dispersion medium should be measured

Preferred metadata: shelf life, elapsed time since stock dispersion preparation and dissolved species concentration in stock dispersion

Questions:

(5a). Comments welcomed.

(6). Dissolution Media

Environmental Media	Biological Media
Distilled water	Simulated saliva
Simulated fresh water	Simulated gastric fluid
Simulated moderate hardness	Simulated intestinal (duodenal) fluid
Estuarine	Simulated alveolar
Seawater	Simulated phagysomal
	PBS- phosphate buffered saline
	Gambles Solution

- Dissolution media to be described in 'assay metadata module'

Preferred metadata: there should be a stated purpose for choosing the dissolution medium/media

Questions:

(6a). Are there prominent media missing in this listing? **10 Yes; 8 No**

(6b). Should the media be listed according to pathways, e.g. saliva, gastric, and intestinal for the GI tract?
11 Yes; 6 No; 1 No Response

(6c). Comments welcome

(7). Accounting for Induction Period

- There may be initial lag times in observing dissolution or periods of initially high rates as surfaces roughen (surface are increases) or equilibrium is established with complexants, ligands and other speciation effects.
- Samples with a nanolayer will exhibit two dissolution rates, one for analytes from the coating and a subsequent one for the central core composition.
- The experimental design should consider these points and establish if preliminary testing should be done.

Preferred metadata: (1). initial and final surface images and (2) initial dissolution rates & solution compositions compared to final particle composition and solution compositions.

(7a). Have the primary sources of induction effects been identified? **10 Yes; 8 No**

(7b). Comments welcome.

(8). Apparatus/Techniques

Metadata	Batch	Flow through	USP Basket	USP Paddle over disc	USP Flow Through	USP Diffusion
temperature	x	x	x	x	x	x
elapsed time	x	x	x	x	x	x
initial mass of solid and medium volume	x		x	x		
initial and final medium pH	x		x	x		
stirring/shaking/mixing/stagnant (identify method)	x		x	x		
medium refreshment & oxygen control	x	x	x	x	x	x
supply & receiving reservoir pH and composition		x			x	
recirculating or once-through media (identify)		x			x	
test sample holder (membrane, mfr., type, pore size, units)		x		x	x	x
solution sampling (time, analyte concentration)	x	x	x	x	x	x
solution aliquot preparation (filter, filter manufacturer, type, pore size and pore size units or centrifugation time, rpm)	x	x	x	x	x	x
analytical method	x	x	x	x	x	x
final solid mass and composition	x	x			x	x
flow rate and influence of flow rate		x			x	
Basket speed of rotation			x			
rotation speed				x		
accumulation of particles at the bottom				x		
means of adding powder (sinker?)				x		
support disc location				x		
bead size and location					x	
residue on beads after test completion					x	
manufacturer of Franz or Transwell Cells						x
pH and composition of donor and receiver cell media						x

Questions:

(8a). Are there additional techniques not found in the table? **6 Yes; 12 No**

(8b). Are the suggested metadata adequate? **0 Yes; 12 No**

(8c). Additional metadata or suggested deletions or comments welcomed.

(9). Data analysis and modeling

Dissolution profile is preferred to a single elapsed time measurement (has merit for QC or scoping tests) and data analysis should be to describe the dissolution profile. Selection of model depends on the extent of dissolution. Low dissolution rates will exhibit a linear profile (particle surface area does not change), which is often termed zero order kinetics, and higher dissolution rates may lead to a non-linear profile (particle surface area decreases significantly), which is often termed first order kinetics.

It should be noted that dissolution profiles may be particle shape dependent. The surface area of a sphere decreases as dissolution progresses, which is not necessarily so with a cylinder or a plane even of the same chemical composition.

Preferred metadata: model selected; adjustments for shape; stoichiometric relationships between the species measured analytically and the chemical composition in the solid; characteristic dissolution rate and half life ($t_{1/2}$). Model examples are given below:

- Non Mechanistic (USP practice)
  - Higuchi (diffusion model)
  - Korsmeyer Peppes (semi-empirical/diffusion)
  - Weibull (empirical model)
- Model-Independent (USP practice)

Zero Order	$Q_t = Q_0 + k_0 \cdot t$
First Order	$\ln Q_t = \ln Q_0 + k_t \cdot t$
Higuchi	$Q = Q_0 + k \cdot t^{0.5}$
Korsmeyer Peppas	$Q = k \cdot t^n$
Weibull	$\log[-\ln(1\text{-}Q)] = b \cdot \log(t - T_i) - \log a$
Model Independent Difference	$f1 = (\sum \lvert R_t - T_t \rvert / \sum R_t) \cdot 100$ (sum from t=1 to n)
Model Independent Similarity	$f2 = 50 \cdot \log\{[1 + (1/n)\sum (R_t - T_t)^2]^{-0.5} \cdot 100\}$

Q is the fraction dissolved at t=0 and time t; k, a, and b are constants; T_i is a lag time; n is the number of time points; Rt and Tt are the amount dissolved of the reference (R) and test (T) materials at time t.

There is a hydrodynamic boundary layer surrounding the dissolving particle, which leads to a diffusion-limited dissolution rate. Changing the agitation or the flow rate may alter the boundary layer thickness, leading to a change in observed dissolution rates. At one point, the mechanism of dissolution may become limited by surface phenomena: pit density, receding ledge distances, formation of new crystalline facets. Hydrodynamic limitations lead to the equations above. Surface kinetics require more elaborate surface

analysis tools for imaging (SEM/TEM) or for surface species elucidation (Surface-enhanced Raman spectroscopy (SERS); time-of-flight (TOF)-based Inductively Coupled Plasma (ICP) Mass Spectrometers (MS)). Pursuing these points will impose additional metadata requirements.

Questions:

(9a). Are there additional models? **6 Yes; 12 No**

(9b) Should one or more of the models be deleted? **0 Yes; 18 No**

(9c) Comments welcomed.

(10). Conclusion and Final Questions

(10a). Are there any missing categories that should be included beyond those in the questionnaire, e.g. definitions,units, etc/ ?

4 No; several comments; soil/porewater suggested

(10b). Is there a 'best in class' paper in terms of the authors reporting on dissolution rates? **See text**

(10c). Is there a material that has the most complete dissolution data set? **5 nano-Ag; 2 nano-ZnO; 1 nano-CuO**
